# Population genetics, community of parasites, and resistance to rodenticides in an urban brown rat (*Rattus norvegicus*) population

**DOI:** 10.1371/journal.pone.0184015

**Published:** 2017-09-08

**Authors:** Amélie Desvars-Larrive, Michel Pascal, Patrick Gasqui, Jean-François Cosson, Etienne Benoît, Virginie Lattard, Laurent Crespin, Olivier Lorvelec, Benoît Pisanu, Alexandre Teynié, Muriel Vayssier-Taussat, Sarah Bonnet, Philippe Marianneau, Sandra Lacôte, Pascale Bourhy, Philippe Berny, Nicole Pavio, Sophie Le Poder, Emmanuelle Gilot-Fromont, Elsa Jourdain, Abdessalem Hammed, Isabelle Fourel, Farid Chikh, Gwenaël Vourc’h

**Affiliations:** 1 Conservation Medicine, Research Institute of Wildlife Ecology, University of Veterinary Medicine, Vienna, Austria; 2 Joint Research Unit (JRU) Écologie et Santé des Écosystèmes (ESE), Institut National de la Recherche Agronomique, INRA, Agrocampus Ouest, Rennes, France; 3 Joint Research Unit (JRU) Epidémiologie des Maladies Animales et Zoonotiques (EPIA), Institut National de la Recherche Agronomique, INRA, VetAgro Sup, Saint-Genès Champanelle, France; 4 Joint Research Unit (JRU) Biologie Moléculaire et Immunologie Parasitaire (BIPAR), Agence Nationale de Sécurité Sanitaire de l’Alimentation, de l’Environnement et du Travail (ANSES), Institut National de la Recherche Agronomique, INRA, Ecole Nationale Vétérinaire d'Alfort (ENVA), Maisons-Alfort, France; 5 Joint Research Unit (JRU) Centre de Biologie pour la Gestion des Populations (CBGP), Centre de Coopération Internationale en Recherche Agronomique pour le Développement (CIRAD), Institut National de la Recherche Agronomique, INRA, Institut de Recherche pour le Développement (IRD), SupAgro Montpellier, France; 6 Contract-based Research Unit (CBRU) Rongeurs Sauvages–Risques Sanitaires et Gestion des Populations (RS2GP), VetAgro Sup, Institut National de la Recherche Agronomique, INRA, Lyon University, Marcy-L’Etoile, France; 7 Unité Mixte de Services (UMS) 2006 Patrimoine Naturel, Agence Française pour la Biodiversité (AFB), Muséum National d'Histoire Naturelle (MNHN), Centre National de la Recherche Scientifique (CNRS), Paris, France; 8 Virology Unit, Agence Nationale de Sécurité Sanitaire de l’Alimentation, de l’Environnement et du Travail (ANSES), Lyon, France; 9 Institut Pasteur, Biology of Spirochetes Unit, National Reference Center and WHO Collaborating Center for Leptospirosis, Paris, France; 10 Joint Research Unit (JRU) Virology, Agence Nationale de Sécurité Sanitaire de l’Alimentation, de l’Environnement et du Travail (ANSES), Institut National de la Recherche Agronomique, INRA, Ecole Nationale Vétérinaire d'Alfort (ENVA), Maisons-Alfort, France; 11 Joint Research Unit (JRU) Laboratoire de Biométrie et Biologie Évolutive (LBBE), Centre National de la Recherche Scientifique (CNRS), Université Claude Bernard Lyon 1, VetAgro Sup, Marcy-L’Etoile, France; 12 Conseil Départemental Hauts-de-Seine, Parc de Chanteraines, Villeneuve-la-Garenne, Paris, France; University of Pretoria, SOUTH AFRICA

## Abstract

Brown rats are one of the most widespread urban species worldwide. Despite the nuisances they induce and their potential role as a zoonotic reservoir, knowledge on urban rat populations remains scarce. The main purpose of this study was to characterize an urban brown rat population from Chanteraines park (Hauts-de-Seine, France), with regards to haematology, population genetics, immunogenic diversity, resistance to anticoagulant rodenticides, and community of parasites. Haematological parameters were measured. Population genetics was investigated using 13 unlinked microsatellite loci. Immunogenic diversity was assessed for *Mhc-Drb*. Frequency of the *Y139F* mutation (conferring resistance to rodenticides) and two linked microsatellites were studied, concurrently with the presence of anticoagulant residues in the liver. Combination of microscopy and molecular methods were used to investigate the occurrence of 25 parasites. Statistical approaches were used to explore multiple parasite relationships and model parasite occurrence. Eighty-six rats were caught. The first haematological data for a wild urban *R*. *norvegicus* population was reported. Genetic results suggested high genetic diversity and connectivity between Chanteraines rats and surrounding population(s). We found a high prevalence (55.8%) of the mutation *Y139F* and presence of rodenticide residues in 47.7% of the sampled individuals. The parasite species richness was high (16). Seven potential zoonotic pathogens were identified, together with a surprisingly high diversity of *Leptospira* species (4). Chanteraines rat population is not closed, allowing gene flow and making eradication programs challenging, particularly because rodenticide resistance is highly prevalent. Parasitological results showed that co-infection is more a rule than an exception. Furthermore, the presence of several potential zoonotic pathogens, of which four *Leptospira* species, in this urban rat population raised its role in the maintenance and spread of these pathogens. Our findings should stimulate future discussions about the development of a long-term rat-control management program in Chanteraines urban park.

## Introduction

With the exception of Antarctica, rats have invaded most habitats of all the continents [1–3]. The introduction of *Rattus* sp. in natural ecosystems has devastating effects and is linked to the extinction of several endemic animal and plant species, particularly in insular environments [4, 5]. The brown rat (*R*. *norvegicus*) is strongly associated with urban ecosystems [6] and has long been considered a nuisance: it eats and contaminates both human and animal food, damages crops in fields and during storage, and may induce fires by gnawing on electrical wires [7, 8].

On top of being a public nuisance, rats are recognized as a serious threat to public health. They carry zoonotic agents including *Leptospira* sp., *Rickettsia typhi*, *Yersinia pestis*, *Salmonella* sp., and Seoul hantavirus among others [8]. Several characteristics make this synanthropic muridae an ideal candidate for maintaining and transmitting zoonotic pathogens: they live in close proximity to humans [2, 6], they have a huge breeding potential [1], and may live in dense populations [9] that enhances inter-individual contacts [10].

Substantial amounts of time and money are spent yearly to control rat populations. In Western countries, control of commensal rodents relies heavily on the use of anticoagulant rodenticides (antivitamin-K, AVK). Anticoagulant rodenticides were first introduced in the 1950s but resistance rapidly appeared in rat populations [11] and more effective AVK compounds, called “second-generation” anticoagulants, were developed during the 1970s and 1980s. The main mechanism of resistance to AVK in the brown rat is associated with mutations within the gene coding for the vitamin K 2,3-epoxide reductase enzyme (VKOR), located on chromosome 1 in brown rats [12].

Despite the ecological and public health impacts of brown rats, studies in urban areas remain scarce [13–17] and there is a paucity of data describing their population ecology in anthropogenic habitats [6]. The urban landscape presents physical barriers that may reduce rat movements and subpopulation connectivity. Urban brown rats seem to show a strong site fidelity [14] with a relatively small home range. The axial dispersal distance of rats seldom exceeds 45–62 meters on ground [14, 18], probably three to four times more within the sewer system [19]. Understanding and tracking the population ecology of urban rats can provide important insights into factors influencing pathogen persistence, spread, and evolution [17].

Landscape genetics is now recognized as an efficient approach to infer ecological processes in spatial studies of infectious diseases [20]. In particular, it may describe major demographic trends such as population size and dispersal, which play critical roles in epidemiology [21]. In spite of the well documented usefulness of population genetics data, only three studies, to date, have used such an approach on urban *R*. *norvegicus* populations [14, 15, 22].

Within an urban setting, parks provide the highest likelihood of human exposure to pathogens and their associated vectors [23]. Therefore knowledge on rat population structure and home range in the context of urban parks is fundamental for understanding pathogen spread and identifying manageable eradication units.

The main purpose of this study was to characterize an urban brown rat population from a French urban park. The Chanteraines park, Hauts-de-Seine, France, was chosen because frequent rat sightings were reported and prevalent human-rat interactions may present a public health risk. In order to describe the population, we performed the following:

1. Obtained morphological and haematological data of the urban rats in the park;
2. Quantified hepatic AVK residue concentration in the sampled individuals and determined the presence of a genetic resistance to anticoagulant rodenticides;
3. Measured the genetic and immunogenic diversity of the rat population and assessed potential gene flow and migration;
4. Characterized the parasite community and investigated the factors associated with parasite occurrence.

In this paper, we defined "parasites" to be members of the viruses, bacteria, protozoa, fungi, helminths, and arthropods [24]. The choice of the investigated parasites was based on their potential zoonotic capacity as well as field constraints. This study will contribute to a better understanding of the population dynamics of urban brown rat. It will also help in the management and control of this species, particularly in urban contexts.

## Materials and methods

### Study area and trapping methods

The study was conducted in Chanteraines park (82 ha, Hauts-de-Seine department, France, 2.261594, 48.84066 D.D.) which includes a farm, a circus, a horse center, and receives an estimated two millions visitors annually. The park has implemented rat-control measures which prohibit the use of rodenticides.

**Fig 1:**
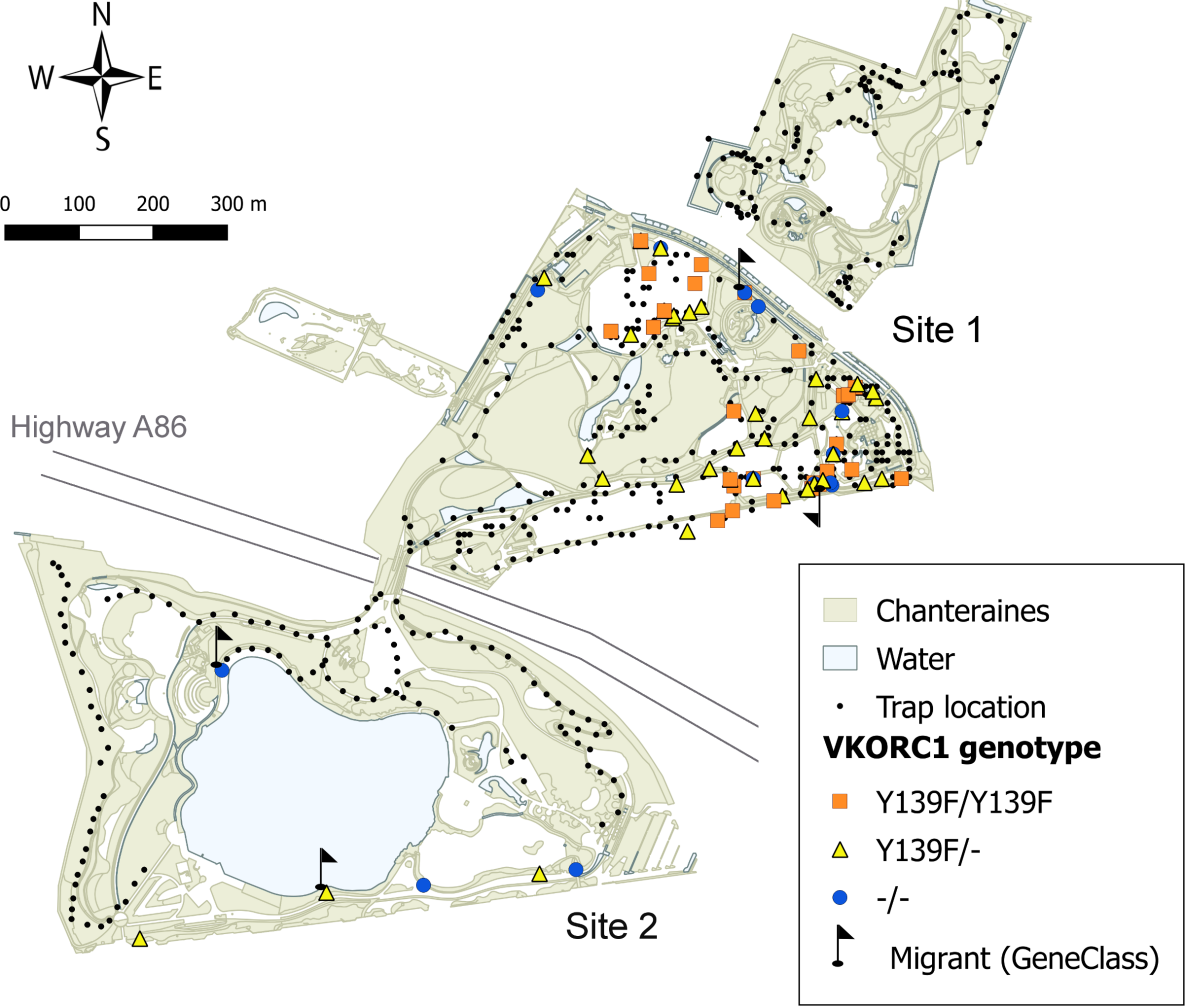
Map of the Chanteraines park (light green: green areas, light blue: water areas). The map shows all trap locations (black dots), highlights location of traps where rats were caught (colored squares, triangles, and dots), specifies the VKORC1 genotype of the captured rats (orange square: *Y139F/Y139F*, yellow triangle: *Y139F/-*, blue dot: *-/-*) and presents results of the GeneClass analysis (flags: migrants).

Because whole rat carcasses were needed for endoparasite research and tissue sampling, trapping-removal methods were used. Trapping was conducted during 12 consecutive days from 10 to 21 January 2011 (the park was closed to the public during this time). Manufrance live-traps (280x100x100 mm) were used, baited with a mixture of peanut butter, oat flakes, and sardine oil [25]. Traps were set in two sites: North (site 1) and South (site 2) (Fig 1). Distance between the centroid of the two sites was approximately 700 m. Traps were spaced 20 m apart whenever possible given physical constraints of the landscape (Fig 1). Geographic coordinates for each trap location were reported from a GPS device (Garmin eTrex®, precision 3–7 m). Since rat sightings were often reported during daytime hours, two trapping periods were organized, i.e. a night-time and a daytime session, covering all 24 hours of the day. Traps were checked twice daily, 07.00–11.00 (rats caught at night) and 18.00–21.00 (rats caught in the daytime), and baited as necessary. For each day and night of trapping, we recorded for each site the number of traps set, rats trapped, and traps sprung (e.g., traps sprung or damaged by people, trapping of non-target species).

### Sample collection

Trapping was conducted in partnership with a private pest control firm during an eradication program conducted in the park. Trapped animals were euthanized by cervical dislocation and blood was collected immediately in the retro-orbital plexus in two 2 ml Eppendorf tubes (Montesson, France): one had no additives, the other was partly filled with EDTA. To take into account the EDTA dilution parameter, EDTA tubes were weighed before and after being filled with blood. Serum fractions were stored at –20°C until use. Peripheral blood smears were done for later examination. Each rat was weighed, sexed, and assigned to one of three age categories: juvenile (< 80 g), subadult (80–180 g for females, 80–200 g for males), or adult (> 180 g for females, > 200 g for males) [26]. Standard body measurements were taken. Ectoparasites (hard ticks, fleas, and lice) were searched macroscopically before necropsy. The spleen, lungs, kidneys, and liver were collected from each rat and frozen at –80°C. Rat digestive tracts were preserved frozen at –20°C for helminth research. A piece of the left hind foot of each rat was stored in ethanol as material for DNA extraction (S1 Fig). All samples were analyzed in 2011, within one year following sampling.

### Haematology and blood smear analyses

White blood-cell, red-blood cell (WBC and RBC, respectively), and platelet counts were measured in total blood samples using the Scil Vet ABC^TM^ Hematology analyzer (Scil Animal Care Company, Gurnee, USA).Giemsa-stained blood smears were examined and the number of *Trypanosoma* sp. per 100 WBC was counted.

### DNA extraction

Genomic DNA was extracted from ethanol-stored foot tissues using silica columns (QIAmp Tissue kit, QIAGEN) for the host genetics component of the study [27].

Molecular detection of parasites was performed on genomic DNA extracts prepared from the different tissue samples using the QiAmp® DNeasy Blood & Tissue Kit (Qiagen, Courtaboeuf, France), according to the manufacturer’s instructions.

### Anticoagulant resistance and AVK residue detection

*Y139F* genotyping was performed using an allele-specific qPCR. A segment encompassing 91 base pairs (bp) was amplified in a Thermocycler Mx3000P (Stratagene, Massy, France) using a reverse primer (5’- TCAGGGCTTTTTGACCTTGTG -3’) which matched the wild type *VKORC1* and the *Y139F* mutated gene and either a forward primer (F_wt_), specific for the wild type exon 3 in rats (5’-CATTGTTTGCATCACCACCTA-3’), or a F_139F_ primer, specific for the *Y139F* mutated gene (5’-CATTGTTTGCATCACCACCTT-3’).

The absence of other missense mutations was verified in ten samples by sequencing the whole gene *VKORC1* as previously described [28] (Biofidal, France). For individuals without *Y139F* mutation, we sequenced the three exons of the *VKORC1* gene to check for other potential mutations.

The liver concentration of eight anticoagulant compounds (brodifacoum, bromadiolone, chlorophacinone, flocoumafen, coumatetralyl, difenacoum, difethialone, and warfarin) was determined using liquid chromatography-mass spectrometry (LC-MS-MS) [29].

### Landscape and immunogenetics

Samples were genotyped using 13 microsatellite loci (*D10Rat105*, *D11Rat11*, *D13Rat21*, *D15Rat64*, *D20Mit4*, *D3Rat159*, *D8Rat162*, *D18Rat11*, *D19Rat62*, *D12Rat49*, *D14Rat110*, *D4Rat59*, and *D5Rat43*) selected from the Rat Genome Database (http://rgd.mcw.edu/). These 13 loci were chosen because they were physically unlinked (i.e. located on different chromosomes), showed high allelic richness, and absence of null alleles in our dataset. Samples were also genotyped using an immune gene (*Mhc-Drb*) and two linked microsatellite loci, hereafter named *D1VKC1A* and *D1VKC1C*. These two loci were chosen for their physical proximity (9,000 and 41,000 bp, respectively) to the *VKORC1* locus. These two markers were expected to provide information about the flow of the resistance allele *Y139F* within the rat population(s) (if different populations share the same haplotype, this may indicate recent gene flow between populations).

Amplification of microsatellite loci was performed in a 10 μl reaction volume containing 1 μl DNA, 5 μl 2X Qiagen Multiplex PCR Master Mix and 0.2 or 0.4 μM of each primer. The Microsatellite Cycling Protocol (Qiagen) was: 95°C for 15 min followed by 40 cycles at 94°C for 30 sec., 57°C for 90 sec., 72°C for 60 sec., and a final extension step at 60°C for 10 min. Genotyping was carried out using an ABI3130 automated DNA sequencer (Applied Biosystems). Alleles were scored using GeneMapper software (Applied Biosystems). Fifteen percent of the samples, randomly chosen, were genotyped twice and the repeatability was 100% for all loci. The *Mhc-Drb* gene was genotyped using the procedure described by Galan *et al*. [30] which allows for retrieval of the sequences from different alleles within each individual.

### Molecular detection of microparasites in tissues

#### Seoul hantavirus

For each animal, RNA was extracted from pooled lung and liver tissue samples using the Qiamp Viral RNA Mini kit (Qiagen, Courtaboeuf, France), according to the manufacturer’s instructions. Specific primers for hantaviruses were used according to the protocol described in Kramski *et al*. [31]

#### Orthopoxvirus

DNA was extracted from lung tissue and orthopoxvirus DNA was then amplified using a PCR protocol described by Ninove *et al*. [32].

#### Coronavirus

RNA was extracted from the lung tissue samples and subjected to RT-PCR using the protocol described by Homberger *et al*. [33].

#### Hepatitis E virus

RNA was extracted from liver tissue using the RNeasy Mini Kit (Qiagen, Courtaboeuf, France) [34] and viral RNA was detected using a nested broad-spectrum RT-PCR as described by Johne *et al*.[35].

#### *Babesia* sp.*/Theileria* sp.

PCR was conducted with the protocol described in Bonnet *et al*. [36] using primers targeting the 18S rRNA gene.

#### *Anaplasma* sp. and *Ehrlichia* sp.

PCR was conducted on DNA extracted from spleen samples using the method of Parola *et al*. [37].

#### *Borrelia burgdorferi* s.l.

PCR was conducted on DNA extracted from spleen samples using the method described in Marconi *et al*. [38].

#### *Bartonella* sp. culture

To specifically isolate *Bartonella* sp., spleen tissue was ground in PBS, then 100 μL of the homogenate was directly plated onto sheep blood agar plates and incubated at 35°C in a humidified atmosphere with 5% CO_2_ for up to 45 days. The plates were assessed daily from day 7 to day 30 before the culture was deemed negative (i.e. absence of colony in the absence of contamination).

#### *Bartonella* sp. PCR

PCR was performed on DNA extracted from spleen samples using primers specific to *Bartonella* sp. [39]. Sequencing was performed in 2017 on newly amplified DNA using primers *pap31* following the protocol described in Michelet *et al*. [40]. Sequencing was conducted at ANSES-Animal Health Laboratory-BIPAR Unit-Vectotiq Team, Maisons-Alfort, France. Sequences were aligned in GenBank using the basic local alignment search tool (BLAST: http://www.ncbi.nlm.nih.gov/BLAST) for the characterization of the species.

#### *Rickettsia* sp.

PCR was performed on DNA extracted from spleen samples using the protocol of Regnery *et al*. [41].

#### Francisella tularensis

PCR was conducted on DNA extracted from spleen and lung samples following Higgins *et al*. protocol [42].

#### *Leptospira* sp.

A partial sequence of the *rrs* gene was amplified by PCR using Taq polymerase (GE Healthcare, Buckinghamshire, UK) and primers A/B under standard conditions [43]. Sequencing was performed at the Genotyping of Pathogens and Public Health Platform (Institut Pasteur, Paris, France) using primers C/RS4 [44]. Sequences were aligned in GenBank using the basic local alignment search tool (BLAST: http://www.ncbi.nlm.nih.gov/BLAST) for the characterization of *Leptospira* genospecies.

### Nucleotide sequence accession numbers

*Leptospira* sp. nucleotide sequences have been deposited in GenBank under accession numbers MF278906 to MF278922. *Bartonella* sp. nucleotide sequence can be found under accession number MF360011.

### Macroparasite collection and identification

Ectoparasites (hard ticks, fleas, and lice) were searched macroscopically, before necropsy, on 83 out of the 86 individuals. Specimens were manually collected, counted, and pooled for later morpho-anatomical identification [45]. Acarid mites were not analyzed. Helminths from the general body cavity were searched macroscopically. In 80/86 rats, helminths were collected from the digestive tract (from esophagus to rectum) under binocular lenses (x6 magnification). Up to 100 helminths were counted individually. If the helminth count was >100, the whole organ content was put into a Petri dish, worm count was conducted on half of the surface and the number was doubled to get the total number of worms in the organ. Helminth identification was based on morphological analyses of drawings using a light microscope equipped with a *camera lucida* (x40 –x100 magnification). If the number of helminths was < 30 per host, then all helminths present were individually identified, a subsample was used if the helminth count was > 30 per host. Nematode species identification referred to Ribas *et al*. [46], del Rosario Robles *et al*. [47], Durette-Desset [48], Hugot and Quentin [49], and Anderson *et al*. [50]. Cestode identification used Casanova *et al*. [51], Makarikov and Tkach [52], Gardner [53], and Khalil *et al*. [54]. Trematode identification relied on to Pojmanska [55] for specimens assigned to the genus *Brachylaima*

### Data analysis

A day and a night trap effort was calculated for each site. The total trap effort (number of traps set per day or night) was adjusted according to the method described by Nelson and Clark [56]. Trap success was calculated to evaluate relative rat abundance during day time and at night on each site using the index described by Theuerkauf *et al*. [57]. Maps were compiled using QGIS v.2.16.3 [58]. All statistical analyses were conducted using R v.3.2.2 [59] and the level of significance was set to 0.05. Data were expressed as absolute frequencies for the qualitative variables and as medians for the quantitative variables. Correlations between quantitative variables were estimated using Spearman´s rank correlation tests. Mean differences between groups were assessed using the Wilcoxon rank-sum test. Differences in proportions were assessed using the two-sample test for proportions. The total parasite species richness (the number of parasite species found in a host species) and the mean parasite burden (mean number of parasites per host) were calculated.

#### Landscape and immunogenetics

For microsatellites and the immune gene (*Mhc-Drb*), we calculated allelic diversity (*A*), observed heterozygosity (*H*_*0*_), Nei’s unbiased expected heterozygosity (*H*_*e*_) [60], and Weir & Cockerham’s inbreeding coefficient (*F*_IS_) [61]. Significance of *F*_IS_ (excess or deficit in heterozygotes) was assessed using 1,000 permutations of alleles. Calculations and tests were conducted using Genetix v.4.05.2 [62]. The genetic structure of the sample was investigated using correspondence analysis adapted to individual diploid genotypes implemented in the Genetix software [62]. Genotypic linkage disequilibrium (LD) between pairs of unlinked microsatellite loci was tested using the Markov chain method implemented in Genepop v.4.2 [63]. We corrected for multiple testing using the False Discovery Rate (FDR) approach [64] implemented in R v.3.2.2 [59]. Bayesian estimates of genetic clustering probabilistically assigns individuals to populations defined by allele frequencies at multiple loci and were determined using Structure v.2.3.4. [65] for 5.0 × 10^4^ burn-in repetitions and 1.0×10^6^ MCMC simulations at five iterations. This allowed for an estimate of the number of genetic units, *K*, using Structure Harvester Web v.0.6.94 [66].

We also conducted statistical analyses at a fine spatial scale using spatial autocorrelation of genetic relatedness. We used Wang´s relatedness coefficient [67] for its high accuracy (low bias) and high precision (low variance). For each distance interval, significance was assessed using permutation tests implemented in the software Spagedi v.1.2 [68]. Finally, we looked for first-generation migrants using assignment tests in GeneClass2 [69]. We computed for each individual its likelihood of belonging to the Chanteraines population (i.e. the *L*_h_ statistics) as recommended when all sources for immigrants have not been sampled [70] using the frequency method. For each individual, we inferred its probability of being a resident using a Monte Carlo resampling procedure. Individuals with a probability lower than 0.01 were excluded as resident. Null allele frequency was estimated using the method of Dempster in FreeNA software [71].

Bottleneck v.1.2.02 [72, 73] was used to test whether the population had experienced a recent bottleneck. A two-phase model (TPM) was assumed with 70% of mutations as conforming to a stepwise mutation model (SMM) and 30% to a multi-step model. Variance was set at 10% and the number of replications at 10,000. Significance of heterozygosity excess over all loci was determined with a two-tailed Wilcoxon sign rank test.

#### Community of parasites

To characterize multiple parasite relationships and to detect whether the parasites were significantly associated, a network approach was used [74, 75]. To test whether the presence of one parasite was independent from the presence of another parasite, a Chi-squared test of independence was performed. The graphical representation of the structuring parasites was realized using the *graph*.*density* connectance function within the *igraph* package [76].

#### Modeling parasite occurrence

To model the occurrence (i.e. presence/absence) of the most prevalent parasites in *R*. *norvegicus* in Chanteraines, generalized linear models (GLMs) were fitted using the R function *glm(*, *family = “binomial”)* within the *glm2* package, using default parameters. For use in model 1, explanatory variables were transformed into categorical variables as follows: site of capture (site 1/site 2), sex (male/female), and body weight alive (converted in a two-categorical variable, < 200 g or ≥ 200 g, as proxies for young and adults, respectively [26]); to identify non-linear effects, body length and hematologic variables (WBC, RBC, platelet counts) were categorized into tertiles. A second model (model 2) was run using the same explanatory variables but the age variable (juvenile, subadult, and adult, as defined above) replaced the body weight alive variable.

Individuals with missing data for one or more variables were excluded from the analysis. When the contingency table between the dependent variable and an explanatory variable presented a zero cell, the explanatory variable was not included in the GLM. The best-fitted model was identified using a stepwise backward selection based on the Akaike Information Criterion (AIC) with the R function *stepAIC(*, *direction = "backward")* within the *MASS* package. To identify risk factors associated with each modeled parasite occurrence, odds ratios (ORs) and 95% confidence intervals (95% CIs) were examined. The OR was considered not significant when the 95% CI included the value 1.

### Ethics statement

This project was run in the framework of a rat control program decided and organized by the management team of the park. This study was not considered to be an “experimental procedure” as defined by the French legislation (Rural Code, Article R214–89) and was therefore not subject to an ethical committee approval in France. The CBGP laboratory received approval (no. B34–169–003) from the Departmental Direction of Population Protection (DDPP, Hérault, France) for the sampling of rodents and the storage and use of their tissues. This study complied with the ethical standards of European regulations governing the care and use of animals in research [77] and it did not involve any endangered or protected species, or protected areas (S1 Checklist).

## Results

Up to 376 traps were set in site 1 and up to 325 in site 2, although the number of traps set varied throughout the study due to field constraints. Trap effort on site 2 was reduced after the fifth day due to low trapping success. Human and material resources were then reallocated to site 1 (S2 Table). A total of 86 rats, all *R*. *norvegicus*, were caught in the Chanteraines park (Fig 1), including 49 (57%) males (one animal with missing data on sex). The number of rats trapped on site 1 was 80, 6 on site 2 (Fig 1). Results per site and sex are summarized in S1 Table. The trap effort during daylight hours was 3,287 and 1,243.5 trap-days in site 1 and 2, respectively. At night time, the trap effort was 3,802 and 1,679 trap-nights in site 1 and 2, respectively. In both sites the trap success was higher during day time (1.58 and 0.32 rats per 100 trap-days in site 1 and 2, respectively) than at night (0.74 and 0.06 rats per 100 trap-nights on site 1 and 2, respectively) (S2 Table).

### Body measurements

Median body weight alive and carcass weight were 194.5 g and 160.0 g, respectively (four animals with missing data on weight). Median body (from nose to anus) and tail (anus to tip of the tail) lengths were 195 mm and 148 mm, respectively (S1 Table). Based on sex and weight criteria, 16 rats were juveniles (nine males), 20 were subadults (13 males), and 46 were adults (25 males). No significant effect of sex on body weight or length was found. Body weight alive and body length were highly correlated (*rho* = 0.95, p < 0.001).

### Haematology

The median WBC, RBC, and platelet counts (x 1,000/mm^3^), measured in 79 brown rats, were 7.2, 8.4, and 514.0, respectively (S1 Table). No significant effect of sex on WBC or RBC count was found, however, females had a significantly higher platelet count than males (p < 0.01). Weight alive was highly correlated with WBC count (*rho =* 0.33, p < 0.01) and RBC count (0.27, p < 0.05), but not with platelet count.

### Anticoagulant resistance and residues

Absence of the *Y139F* mutation on a *VKORC1* allele is denoted "-". The study of the *Y139F* polymorphism within the *VKORC1* locus showed that 17/86 (19.8%) rats were of the wild type (*-/-*, i.e. homozygous non-mutated), 42 (48.8%) carried a heterozygous mutation (*Y139F/-*), and 27 (31.4%) were homozygous for the *Y139F* mutation (*Y139F*/*Y139F*). Sequencing of *VKORC1* revealed that no mutation was present on *VKORC1* in wild-type rats. The microsatellite loci *D1VKC1A* and *D1VKC1C* (physically linked to *VKORC1* locus) showed six and five different alleles, respectively. The resistance allele *Y139F* of the *VKORC1* gene was in 98% of cases associated with a single microsatellite (*D1VKC1A*: allele 328, *D1VKC1C*: allele 259).

**Fig 2:**
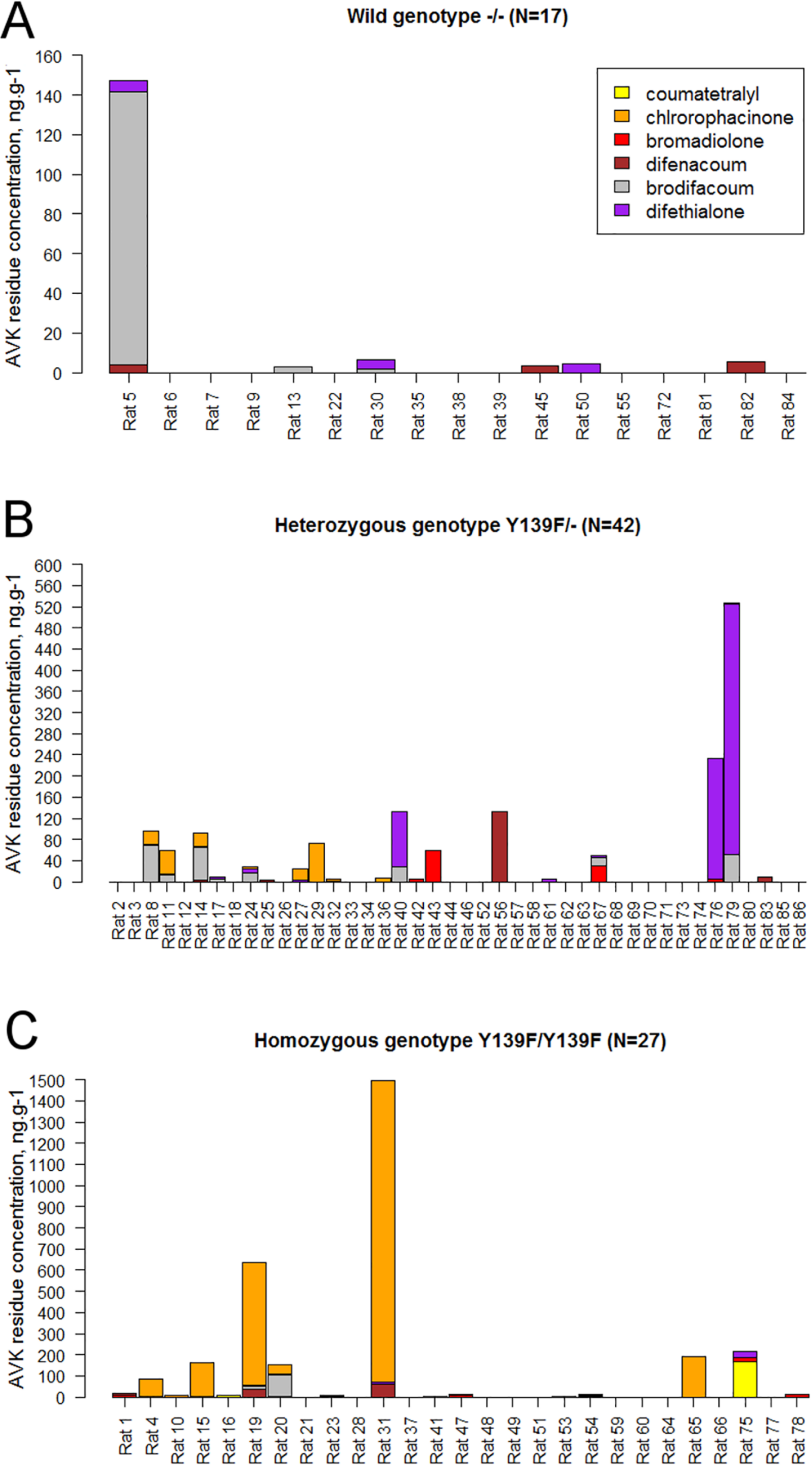
Individual liver concentration (ng.g^-1^) of the eight AVK compounds investigated, depending on *VKORC1* genotype.

**Fig 3:**
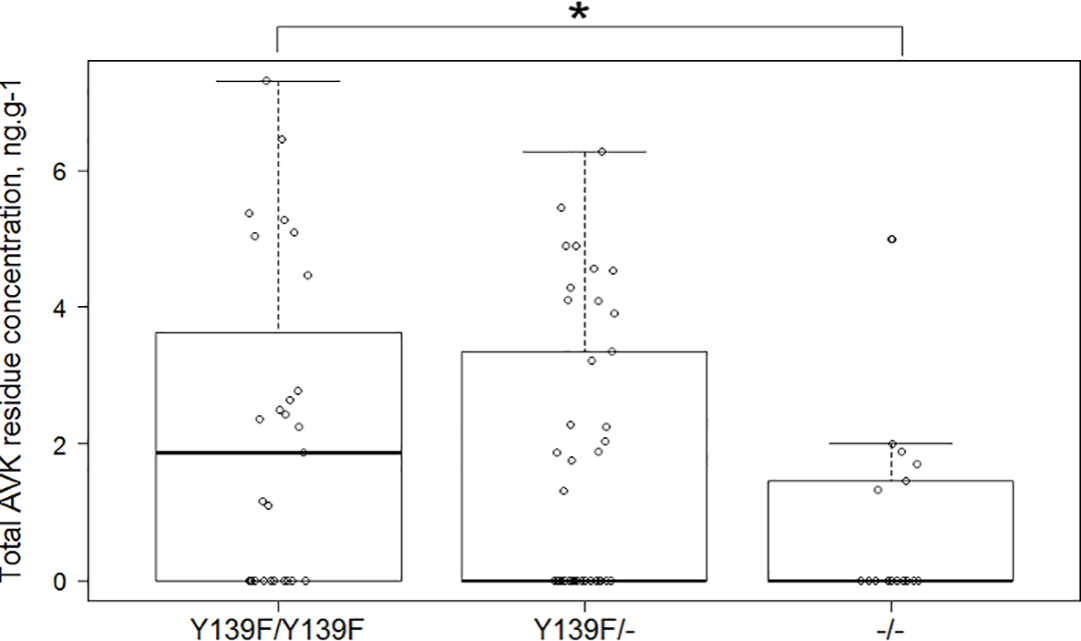
Boxplot and scatterplot of the total AVK residue liver concentration (ng.g^-1^) depending on the *VKORC1* genotype (log scale).

AVK residues were detected in the liver of 41/86 rats (47.7%). Warfarin and flocoumafen were not found in our samples; coumatetralyl residues were found in 2/86 (2.3%) rats; chlorophacinone and bromadiolone were each detected in 10 (11.6%) and 11 (12.8%) samples respectively; difethialone and difenacoum were each detected in 17 (19.8%) samples; brodifacoum was detected in 24 (27.9%) individuals. Seventeen (41.5%), 12 (29.3%), eight (19.5%), and four (9.7%) of the residue-positive rats presented one, two, three, and four different AVK residues, respectively (Fig 2). The median total AVK concentration among the residue-positive individuals was 15.1 ng.g^-1^ (range = 2.0–1,496 ng.g^-1^). AVK residues were found in 6/17 (35.3%) *-/-* individuals (Fig 2A), 19/42 (45.2%) *Y139F/-* individuals (Fig 2B), and 16/27 (59.2%) *Y139F/Y139F* individuals (Fig 2C). Prevalences of AVK residues were independent of the genotype (p>0.05). The median AVK liver residue concentrations were 5.5 (range = 0.0–1,496.0), 0.0 (range = 0.0–527.2), and 0.0 (range = 0.0–147.3) ng.g^-1^ in *Y139F/Y139F*, *Y139F/-*, and *-/-* individuals, respectively. Mean total AVK residue concentrations were significantly different between wild genotype (*-/-*) rats and homozygous resistant (*Y139F/Y139F*) rats (mean = 9.99 and 111.75 ng.g^-1^, respectively, p < 0.05) (Fig 3).

### Landscape and immunogenetics

Microsatellite analysis revealed a low allelic richness, ranging from 3 to 10 depending on the loci (mean 5.6 ± 1.6) (Table 1). The inbreeding coefficient *F*_IS_ was negative for 9 of the 13 (69.2%) microsatellite markers, with only two significant values (markers *D18Rat11* and *D12Rat49*, *F*_IS_ = –0.124 and –0.199, respectively, both p < 0.05). Overall, the rat population was in accordance with Hardy-Weinberg equilibrium criteria, with a slight excess in heterozygotes (*H*_*e*_ = 0.628 ±0.069; *H*_*0*_ = 0.645 ± 0.078).

**Table 1:** Characterization of the 13 microsatellite markers.

	*H*_*e*_	*A*	*F*_*IS*_	*F*_*IS*_ p-value	%95 CI
D10Rat105	0.641	3	0.112	0.933	(-0.04262−0.25938)
D11Rat11	0.666	5	0.075	0.865	(-0.07914−0.22958)
D13Rat21	0.721	6	-0.016	0.450	(-0.15034−0.11109)
D15Rat64	0.587	5	-0.070	0.242	(-0.23880−0.08538)
D20Mit4	0.533	5	-0.004	0.554	(-0.13017−0.12532)
D3Rat159	0.591	5	-0.043	0.344	(-0.19969−0.10505)
D8Rat162	0.657	6	0.062	0.843	(-0.09155−0.19885)
D18Rat11	0.621	7	-0.124	0.022	(-0.22237− -0.03088)
D19Rat62	0.579	4	-0.044	0.351	(-0.21588−0.10860)
D12Rat49	0.572	5	-0.199	0.011	(-0.35414− -0.05770)
D14Rat110	0.793	10	-0.056	0.175	(-0.14642−0.02536)
D4Rat59	0.615	6	0.036	0.746	(-0.09691−0.16591)
D5Rat43	0.594	6	-0.096	0.126	(-0.21856−0.02092)

*H*_*e*_: expected heterozygosity, A: allelic diversity, *F*_*IS*_: inbreeding coefficient (*F*_*IS*_) with its p-value, %95 CI: 95% confidence interval.

The microsatellite dataset showed a high degree of LD (47%). The correspondence analysis (S2 Fig) revealed a homogeneous genetic structure of the Chanteraines rats. Bayesian analysis showed a sharp increase of mean LnProb from *K* = 1 to *K* = 5 (S3 Fig). This result suggests the existence of one single cluster (*K* = 1) in this system, associated with low level of genetic substructure and consequently high levels of gene flow. Four outliers were detected (all males and randomly distributed within the park, Fig 1). The GeneClass analysis revealed that these four individuals had a low probability of being residents of the Chanteraines rat population (p < 0.01).

**Fig 4:**
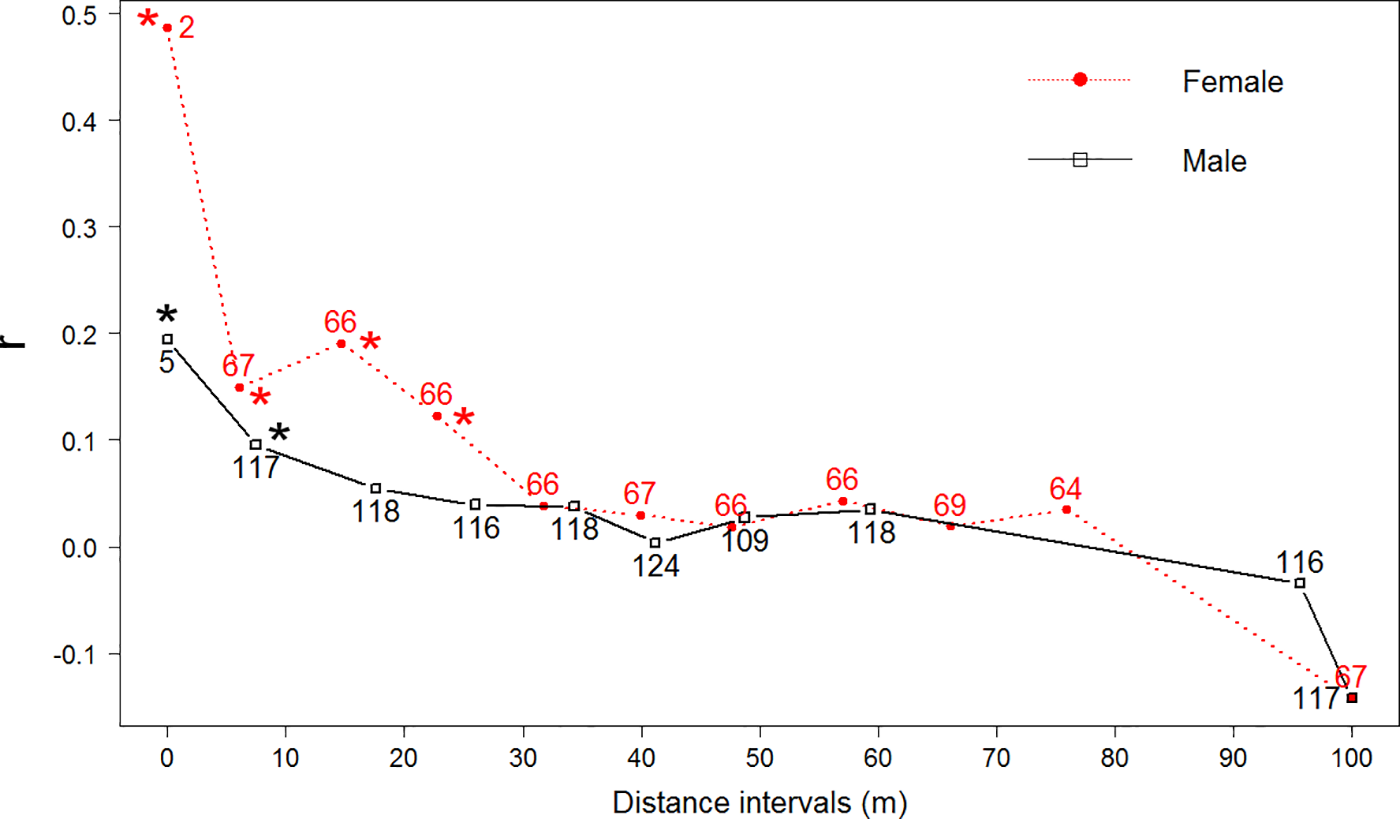
Pairwise relationship coefficients between female and male individuals (*r*) plotted against geographic separation (in meters, m) in Chanteraines rat population assessed using 13 microsatellite markers. The sample consisted of 49 males and 37 females; sample size at each distance is specified. Coefficient values significantly different from zero are shown with an asterisk.

The spatial autocorrelations of genetic relatedness showed that the rat population was highly structured at a small spatial-scale. We found significantly higher levels of relatedness (p < 0.05) between geographically closer individuals. Relatedness values were systematically higher for females than for males for each distance interval considered (Fig 4). This difference in significance was not due to sample size because sample size was high for both genders, allowing a good assessment of the relatedness for each interval. Finally there was no evidence of a recent bottleneck in the population (p = 0.45).

The 454-sequencing of the immune gene *Mhc-Drb* exon 2 revealed five alleles, two of which were highly dominant in the population (frequency >30%) while the others were rarer (frequency < 15%). *F*_IS_ value was very high (0.352; p < 0.0001). This deficit was not associated with one particular allele, as expected for a positive selection on a hyper favorable allele, but was most likely due to a null allele (whose frequency was estimated at 0.14 using FreeNA software).

### Parasite detection

#### Microparasites

**Fig 5:**
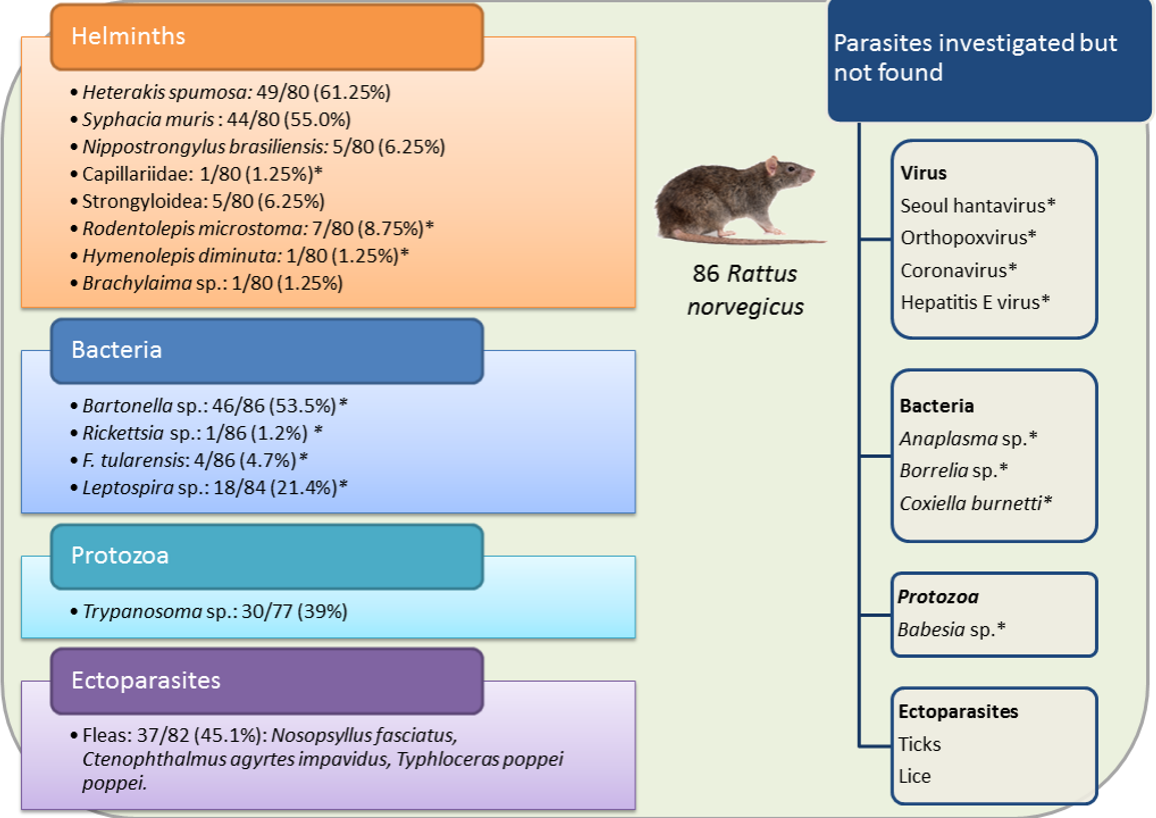
Summary of the parasites investigated and detected in the brown rat population from Chanteraines public park, France. Asterisks show potentially zoonotic parasites.

PCR analyses showed no evidence of infection by Seoul hantavirus, orthopoxvirus, hepatitis E virus, coronaviruses, *Babesia* sp., *Anaplasma* sp., or *Borrelia* sp. in the brown rats sampled in the Chanteraines park (Fig 5). The prevalence of *Bartonella* sp. estimated by culture was 58.2% (32/55, 31 individuals with missing data) whereas it was 31.4% (27/86) when estimated by PCR. A rat was considered *Bartonella*-positive if it showed a positive culture and/or positive PCR, resulting in an overall prevalence of *Bartonella* sp. equal to 53.5% (46/86). Only 11 newly amplified DNA sequences enabled to obtain good quality sequencing data in 2017. The other DNA templates were too low-quality, most likely due to the degradation of the DNA stored since 2011. Sequences of all eleven 107-bp products were identical and had 100% identity with *B*. *henselae* sequence. The prevalence of *Rickettsia* sp. was 1.2% (1/86); the DNA sequence retrieved in 2017 was too low-quality for sequencing. The prevalence of *F*. *tularensis* DNA was 4.7% (4/86). The prevalence of *Leptospira* sp. DNA in kidney tissue was 21.2% (18/85). Five *Leptospira* genospecies were identified: the most prevalent was *L*. *interrogans* (8/18, 44.4%), followed by *L*. *borgpetersenii* (6/18, 33.3%), *L*. *broomii* (2/18, 11.1%), *L*. *santarosai*, and *L*. *kirschneri* (both 1/18, 5.6%). The prevalence of *Trypanosoma* sp. was 39% (30/77, 9 individuals with missing data) but among the infected rats, the trypanosome count varied from 3 to >150 per 100 WBC, with [0–25] trypanosomes per 100 WBC in eight rats, [25–50] in eight rats, [50–75] in two rats, [75–150] in three rats, and >150 in nine rats (Fig 5).

#### Macroparasites

The research for helminths was conducted on 80 *R*. *norvegicus* (Fig 5). No parasite was found in the general cavity and only one individual presented an unidentified immature nematode in the stomach. A total of six helminth species were found: *Heterakis spumosa* was found in the large intestine of 49 (61%) rats, *Syphacia muris* in the caecum of 44 (55%) rats, *Nippostrongylus brasiliensis* in the duodenum of five (6%) rat, a worm of the family Capillariidae was found in the duodenum of one rat, and five rats had stage 3 larvae of Strongyloidea. Three species belonging to Platyhelminthes were recovered: two tapeworms in the small intestine, *Rodentolepis microstoma* in 7 (9%) rats and *Hymenolepis diminuta* in one rat, and five flukes (*Brachylaima* sp.) in one rat. The helminth species richness in *R*. *norvegicus* in Chanteraines park was nine. The number of observed helminth species per host varied from zero to three (median = 1.0).

No ticks or lice were found. Prevalence of flea infestation was 45% (37/82) (Fig 5), with a median number of two fleas per individual (range 1−26). Of 130 fleas collected, 111 were identified. They were distributed within three species: 99 (89.1%) were *Nosopsyllus fasciatus*, 11 (10%) were *Ctenophthalmus agyrtes impavidus*, and one female (0.9%) was *Typhloceras poppei poppei*.

### Parasite species richness and associations

**Fig 6:**
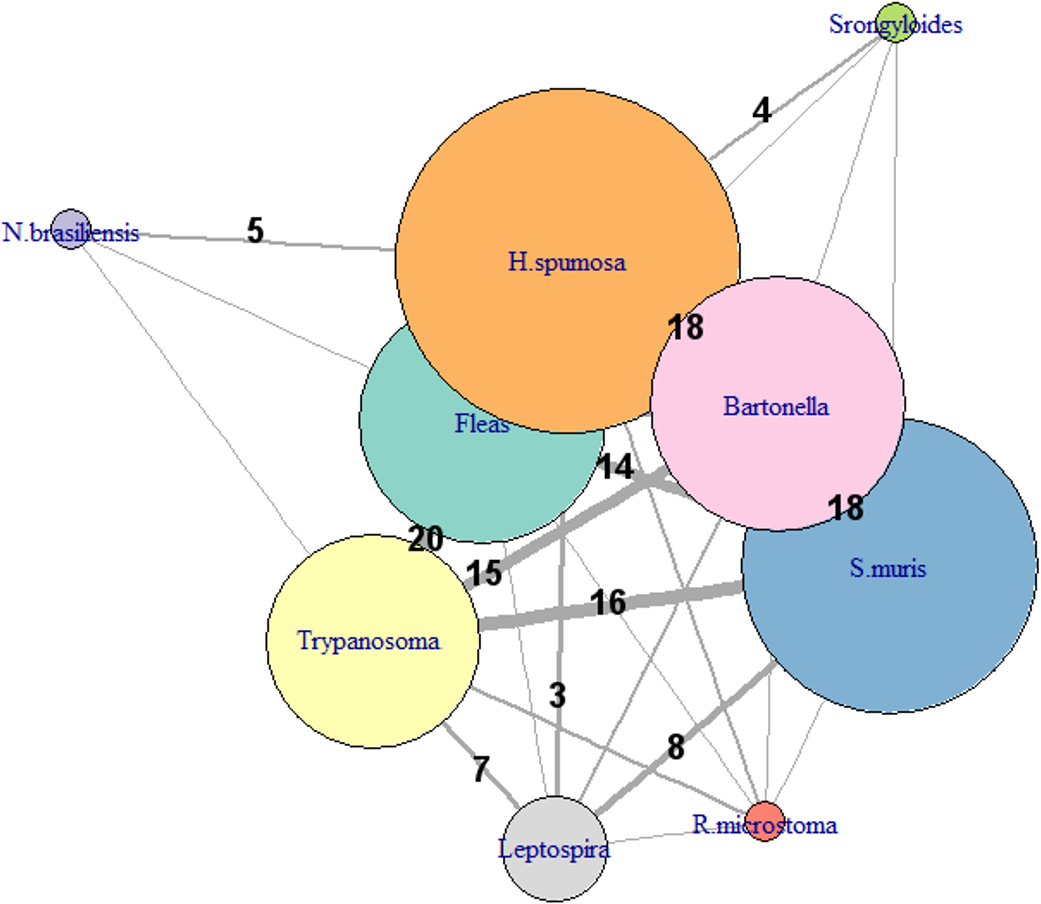
Graphical representation of the structuring of the main parasites of the brown rats from Chanteraines using the network approach. The edge width is proportional to the number of individual hosts involved (numbers correspond to the number of rats sharing the parasites). The node size is proportional to the number of hosts infected (prevalence).

The total parasite species richness in *R*. *norvegicus* in Chanteraines was 16 (Fig 5). There was no evidence of parasites in two (2.5%) rats among 80 screened for all parasites. Seventy rats (87.5%) carried more than one parasite, among them, the mean parasite burden was 3.3 ± 1.0 (range = 2–6). The parasite burden was not significantly correlated with weight (p > 0.05). There was no significant difference in the parasite burden between males and females, neither between migrants and non-migrants. The network analysis, conducted on 66/86 (76.7%) brown rats with no missing data, revealed no significant overall parasite association (observed connectance: 0.81, p = 0.80) (Fig 6) but infestation with *S*. *muris* was significantly associated with infestation with *H*. *spumosa* (p < 0.05).

### Modeling parasite occurrence

GLMs were fitted on 66 individuals to model the occurrence (presence/absence) of the five most prevalent parasites, i.e. fleas (all species considered), *Trypanosoma* sp., *S*. *muris*, *H*. *spumosa*, *Bartonella* sp., and *Leptospira* sp. (Fig 5). Body weight and body length were highly correlated (see above), therefore body length was not included in the models.

With regard to model 1, the stepwise backward selection identified a best-fitted model in the *S*. *muris*, *H*. *spumosa*, *Bartonella* sp., and *Leptospira* sp. occurrence models but did not identify a best-fitted model for flea and *Trypanosoma* sp. occurrence using the input variables. None of the four best-fitted submodels 1 included the variable *platelet count* (Table 2). After model selection, the best-fitted submodel 1 for *S*. *muris* occurrence included the variables *site*, *sex*, and *weight*. The risk factor analysis showed that rats in site 2 and rats ≥ 200 g had a significantly lower risk of *S*. *muris* infestation while males had a significantly higher risk than females. The variables retained in the best-fitted submodel 1 of *H*. *spumosa* occurrence were *sex*, *weight*, and *WBC count*. The risk factor analysis showed that rats with WBC count [6.6–9.2] x1,000/mm^3^ had a significantly higher risk for *H*. *spumosa* infestation. Regarding *Bartonella* infection, the variables *WBC* and *RBC count* were retained in the final submodel 1. The risk factor analysis showed that rats with WBC count ≥ 6.6 x1,000/mm^3^ presented a significantly lower risk of *Bartonella* infection (coefficient of *RBC* variable was not significant). The best-fitted submodel 1 for *Leptospira* occurrence retained the single variable *WBC count* and the risk factor analysis showed that a WBC count ≥ 9.2 was significantly associated with a lower risk of *Leptospira* infection (Table 2).

**Table 2:** Model 1 results. Adjusted odds ratios and 95% confidence intervals for pathogen occurrence in *R*. *norvegicus* from Chanteraines park calculated using the best-fitted general linear model identified using a stepwise backward selection on the Akaike Information Criterion.

Variables	Categories	*S*. *muris*	*H*. *spumosa*	*Bartonella* sp.	*Leptospira* sp.
Site	Site 1	Ref	/	Not included	Not included
	Site 2	0.07 (0.00–0. 62)*	/	Not included	Not included
Sex	Female	Ref	Ref	/	/
	Male	5.22 (1.58–21.03)*	0.08 (0.26–2.75)	/	/
Body weight alive (g)	< 200	Ref	Ref	/	/
	≥ 200	0.15 (0.04–0.52)*	3.41 (0.99–12.92)	/	/
WBC count (x 1,000/mm^3^)	< 6.6	/	Ref	Ref	Ref
	[6.6–9.2]	/	8.52 (2.06–43.23)*	0.17 (0.02–0.82)*	0.67 (0.17–2.56)
	≥ 9.2	/	3.23 (0.81–13.89)	0.19 (0.02–0.94)*	0.10 (0.00–0.63)*
RBC count (x 1,000/mm^3^)	< 7.0	/	/	Ref	/
	[7.0–8.8]	/	/	4.86 (0.97–37.65)	/
	≥ 8.8	/	/	4.08 (0.83–31.00)	/
Platelet count (x 1,000/mm^3^)	< 452.0	/	/	/	/
	[452.0–645.0]	/	/	/	/
	≥ 645.0	/	/	/	/

No model can be fitted for flea and *Trypanosoma* sp. occurrence with the explanatory variables considered. “Not included” means that the variable was not included in the GLM because the contingency table between the dependent variable and an explanatory variable presented zero cells.

*Significant adjusted odds ratio are shown by asterisks.

Results for model 2 (using age categories instead of the two-categorical variable *body weight alive*) were very similar to model 1 except that a model 2 could be fit to flea occurrence. The submodel 2 of flea occurrence retained *sex* and *WBC* variables, and rats with WBC count [6.6–9.2] x1,000/mm^3^ had a significantly lower risk for flea infestation. No model 2 could be fitted for *Trypanosoma* sp. and *Bartonella* sp. occurrence (S3 Table).

## Discussion

Through the comprehensive screening of 86 rats captured in an urban park near Paris, we showed that the population received migrants and presented a high frequency of genetic resistance to AVK rodenticides. Nearly half of the rats trapped, independently to the resistance genotype, presented liver residues of AVK rodenticides. Almost all individuals (97.5%) were infected with at least one parasite.

### Haematological parameters

To the best of our knowledge, there are no haematological reference values for wild brown rats. WBC, RBC, and platelet counts of the sampled Chanteraines rats were within the range of published reference values on laboratory brown rats [78, 79]. Like laboratory brown rats, our sampled individuals showed variations in haematological values depending on age (approximated using body weight alive) and sex [78, 79].

### Landscape and immunogenetics

Our results suggest that there was a spatial connection and potential gene flow between the rats in Chanteraines park and undescribed surrounding rat population(s). This hypothesis is supported by the high degree of LD between microsatellite loci and a relatively high genetic diversity (*H*_*e*_ = 0.628 ± 0.069). Increased linkage disequilibrium can originate from a recent mixing of individuals from several subpopulations that have different allele frequencies, a spatial structuring into several subpopulations, or a bottleneck event [80]. Based on genetic analyses, there was no evidence of a recent bottleneck or substructuring into several subpopulations in our sample. Accordingly, we suggest that LD may have been caused by a recent colonization of the park by genetically differentiated subpopulations followed by high mixing of individuals from these populations. As noted in Slatkin *et al*. [80], the mixing of individuals from different subpopulations that have different allele frequencies creates LD and the decay of LD under recombination alone can be greatly retarded.

High genetic diversity was unexpected considering the small area of study, the strong site fidelity, the aggregation of related individuals, and the small dispersion range typical of urban *R*. *norvegicus* [14, 81]. The genetic diversity of the Chanteraines population was nevertheless similar to what has been described in urban rat populations from Baltimore, USA (*H*_*e*_ = 0.658–0.780) [14] and Salvador, Brazil (0.57–0.72) [15]. There was an excess of heterozygotes (negative *F*_IS_ values in 9 out of 13 microsatellite markers) indicating probable outbreeding [82]. Furthermore, four first-generation migrants were detected, all males, most likely coming from outside Chanteraines park. Fine spatial-scale analysis of the genetic relatedness showed that the population was spatially structured in small demes of related females. These data indicated differential dispersal behavior between sexes, with females dispersing less than males at smaller distances (< 30 m). This strongly suggests a male-biased dispersal and female philopatry, in accordance with other genetic [14, 15] and ecological studies [83, 84] on brown rats.

*F*_*IS*_ value at the *Mhc-Drb* locus was very high (while *F*_*IS*_ calculated at microsatellite loci was negative), which proved a deficit in heterozygotes at *Mhc-Drb* locus, likely originating from the presence of a null allele at this locus. Allelic diversity at the *Mhc-Drb* immune gene and microsatellite loci was low. The low polymorphism observed at *Mhc-Drb* may imply lower resistance to pathogens [85, 86] and may relate to the high parasite diversity and burden in this rat population. We lack comparison data on other urban brown rat populations, however *Rattus* sp. from Southeast Asia, the area of origin of *Rattus* sp. [2], had a much higher genetic diversity in *Drb* genes and microsatellite loci [30, 87]. Reduction in neutral (microsatellite loci) and adaptive (*MHC* loci) genetic diversity may originate from bottleneck events. It can also derive from one or several successive founder events (i.e. when a small number of individuals found a new population in a new area) and can occur in fragmented, small, isolated populations (such as urban populations which typically live in a fragmented habitat [88]) where substantial genetic drift induces reduced genetic diversity [85, 89].

### Genetic resistance to AVK and toxicology

Despite the prohibition of AVK rodenticide usage in Chanteraines park, we found a high prevalence (55.8%) of the mutated allele *Y139F* and AVK rodenticide residues in nearly half of the sampled individuals. The mutated *Y139F* locus was highly linked with a single microsatellite allele at both loci *D1VKC1A* and *D1VKC1C* which strongly supports the hypothesis of regular gene flow between rats in the Chanteraines park, which most probably constitute one single population from a genetic point of view. The *Y139F* mutation is widely spread in France [12, 28]. Specifically, it has been described in four departments near Hauts-de-Seine [28], where Chanteraines park is located. Therefore, we suppose that the mutated allele *Y139F* may be regularly introduced in the Chanteraines population by migrants from nearby sites.

Six out of the eight AVK residues investigated were retrieved in the sampled rats (bromadiolone, difenacoum, brodifacoum, chlorophacinone, coumatetralyl, and difethialone). This suggests a regular immigration of rats from neighborhoods (where rodenticides are in use). More probably, our findings indicate a usage of AVK rodenticides in the park, in spite of the ban. Warfarin and flocoumafen are not marketed in France and, as expected, no residues of these molecules were detected. AVK concentrations were widely variable, from 2 ng.g^-1^, the lower limit quantifiable by the method, to 1,425.3 ng.g^-1^. Most rats (30/41, i.e.73.2%) presented a total AVK residue liver concentration below the threshold of 100 ng.g^-1^, considered as the lowest estimate for a no-effect concentration on blood coagulation in susceptible rats. Because second generation AVK can persist several months in rat liver [90], high frequency of hepatic AVK residues can most likely be explained by a past exposure to rodenticides. Only three rats showed liver residue concentration > 100 ng.g^-1^, which is capable of inducing a toxicological effect. Two *Y139F/Y139F* homozygous rats presented a difethialone concentration of 1,425.3 and 580 ng.g^-1^. One heterozygous rat *Y139F /-* presented a brodifacoum liver concentration of 472.1 ng.g^-1^. These three animals had likely ingested an AVK compound just before being trapped.

### Parasite diversity and community of parasites

Sixteen different parasite genera were retrieved in this rat population, including eight helminth species, three flea species, one protozoan, and four bacterial genera (*Bartonella* sp., *Rickettsia* sp., *Francisella* sp., and *Leptospira* sp.). The mean parasite burden was 3.3 parasites per host, with 87.5% of the sampled rats carrying at least two parasites. These results reveal high species richness in parasites of *R*. *norvegicus* and confirm that co-infection in hosts is the rule, not an exception [91, 92]. Furthermore, the presence of several potentially zoonotic pathogens (*R*. *microstoma*, *H*. *diminuta*, *Bartonella* sp., *Rickettsia* sp., *F*. *tularensis*, *Leptospira* sp.) raises the role of urban rats in the maintenance and spread of potentially zoonotic pathogens to humans and to other animals in Chanteraines.

The two most prevalent parasites were the worms *H*. *spumosa* (61.2% of rats infested) and the rat pinworm, *S*. *muris* (55.0%). Statistical evidence of a significant association between these two intestinal worms was found. *Syphacia muris* is primarily a rat-to-rat transmitted species [93] whereas *H*. *spumosa* is a parasite of rats, mice, and occasionally hedgehogs [94]. Similarities in the life cycles and transmission routes of these helminths can lead to their co-occurrence in the same host. For both species, infestation occurs through oral ingestion of infective embryonated eggs. However, *H*. *spumosa* is more typically a soil-transmitted helminth species (eggs are excreted in the feces) whereas female oxyurid nematode *S*. *muris* deposit its eggs on the perianal region of the host [95, 96]. Even though common factors may drive co-infestation by both species, our models did not show analogous risk factors of occurrence of these two helminths. These findings may reflect that other epidemiological factors influence the infection patterns of the two worms.

*Bartonella henselae*, the agent of the cat-scratch disease, was identified in 11 of the *Bartonella-*positive samples. This species is not commonly described in *Rattus* species, however, a high prevalence of IgG antibodies against *B*. *henselae* (31/342, 9.1%) was reported in *R*. *norvegicus* from Cyprus [97].

*Leptospira* sp. was detected in 21.4% of the individuals and displayed a strikingly high genotype diversity, with five genospecies identified, all pathogenic for humans [98]. To our knowledge, the highest *Leptospira* species diversity was described in the tropical island of Mayotte (Indian Ocean) where four *Leptospira* genospecies have been retrieved in *Rattus rattus* [99]. Other studies reported a low genetic diversity among local *Leptospira* strains [100–102]. Rodents from the genus *Rattus* are considered as the main reservoir of *Leptospira* sp. which they shed in their urine [98]. Further studies are needed to evaluate this public health issue in the Chanteraines public park.

### Modeling parasite occurrence

Our models showed a differential relationship between the investigated risk factors and parasite occurrence, depending on the parasite considered. We were unable to find any common risk factors of parasite occurrence. *WBC count* was included in three out of four best-fitted submodels, suggesting immune cell response to pathogen presence. Both best-fitted submodels for helminth occurrence included *sex* and *body weight* variables, while these two variables were not included in the two submodels for bacteria occurrence.

### Limits

A drawback of the present work is the low number of animals caught, which reduced the statistical power of our analyses. Our trapping effort was high. The field work was conducted by rodent experts, and different baits were tested. However, despite these efforts, the number of captured rats was low. Rats in the Chanteraines park were known to be abundant and many visitors reported rat sightings during the day time. Administrative and financial constraints did limit the time dedicated to field work (the park was closed to the public during 12 days). Prebaiting could have enhanced the trap success but the trapping period was considered too short to include a prebaiting phase. Indeed, rats show neophobic behavior, particularly with unexpected objects [6]. However, if this avoidance is often overcome within a few days when resources are scarce [66, 103], aversion to new food can be exacerbated when resources are abundant [81]. Rats in Chanteraines had access to food provided *ad libitum* to the zoo animals and/or refuse left by visitors. This may explain why rats did not explore the food sources in the traps and thus our low trap success.

### Impacts on practical rat control and public and animal health

We identified seven potential zoonotic pathogens within this urban rat population. The severity of the diseases induced in humans and the risk levels of contamination are pathogen-dependent. A regular surveillance of rodent populations is essential to predict future disease prevalence and to identify emerging rodent-borne diseases. Moreover, some rat-borne parasites can be transmitted to the zoo animals and to domestic dogs (e.g., *Leptospira* sp.), and are therefore of veterinary concern. Rat abundance was lower in site 2 than in site 1 which may induce spatial heterogeneity in the risk of pathogen spill-over events. Trap success was higher during the day versus night, confirming visitor reports of daylight rat sighting, but surprisingly contradicting observations on *R*. *norvegicus*, considered as a nocturnal species mostly active at dusk and dawn [6]. Daytime rat activity increases the probability of encounter, and therefore the probability of direct disease transmission between rats and humans (or domestic animals), which represents an uncommon epidemiological situation [6].

The Chanteraines park is engaged in a program for bird conservation and urban rats are a food resource for protected raptors [104]. For this reason, the use of AVK rodenticides is prohibited in the park. However, the presence of AVK residues in the liver of the rodents suggests a use (even if minimal) of these molecules inside or in the close vicinity of the park. The park receives around two million visitors annually, some come with their pets, raising the risk of poisoning if intensive rodenticide treatment is conducted. On the other hand, an uncontrolled growing rat population arouses health concerns related to the transmission of zoonotic diseases and regular rat sighting may stress and repulse visitors.

A variety of methods are available to manage rodent populations. These methods include physical (e.g., traps, barriers), chemical (e.g., toxic baits, fumigants, repellents), or biological/cultural (e.g., resistant plants, crop type, sanitation, habitat manipulation) approaches [4]. Each method has advantages and disadvantages and a site-specific assessment should be made before implementing a rodent management program. Knowledge on rat biology, ecology, and genetic structure constitute an important foundation for developing an effective rat-control action [105], in particular for the development of an ecologically-based rodent management program [106]. Population genetic analyses showed that the Chanteraines rat population is not closed (i.e. immigration can occur from outside), which indicates that the park will likely be recolonized by new individuals during or after eradication, making eradication programs even more challenging.

Besides being of public health relevance, rats bring stress to residents of infested neighborhoods, give a bad image of the park to visitors, damage property, can spoil animal food, contaminate water with feces and urines, and cause financial loss [7, 8]. Our findings should stimulate future discussions on the development of a long-term rat-control management program in Chanteraines urban park and more globally, in urban green areas [107].

## Supporting information

S1 Checklist ARRIVE checklist. (PDF)

S1 Table Summary of the results per site and sex. Median and range (minimum–maximum) are given for the quantitative data, number and percentage for the qualitative data. One animal on site 1 was not sexed, weight data were missing for two females and two males on site 1, which therefore could not have been categorized as young or adult.
(PDF)

S2 Table Setting and results of the trapping sessions, 10–21 January 2011, on sites 1 and 2 in Chanteraines park (Hauts-de-Seine, France). (PDF)

S3 Table Model 2 results. Adjusted odds ratios and 95% confidence intervals for pathogen occurrence in *R*. *norvegicus* from Chanteraines park calculated using the best-fitted general linear model identified using a stepwise backward selection on the Akaike Information Criterion.
(PDF)

S1 Fig Flow chart illustrating the samples taken on the trapped brown rats, the storing processes and subsequent analyses for each sample. (TIF)

S2 Fig Results of the correspondence analysis (implemented in the Genetix software) of the brown rat population structure in Chanteraines (correspondence analysis was adapted to individual diploid genotypes). Individuals from site 1 are in black, red signs represent individuals from site 2. Identification names of the four individuals outside the main scatter plots are written, they correspond to the four migrants.
(TIF)

S3 Fig Structure Harvester output. Plot of mean likelihood L(K) and variance per K value from Structure (86 individuals genotyped for 13 polymorphic microsatellite loci).
(PDF)

S1 Excel Spreadsheet Dataset. (XLSX)

S2 Excel Spreadsheet Results of the microsatellite genotyping. (XLSX)

## References

[1] Battersby S, Hirschorn RB, Amman BR. Commensal rodents. In: Bonnefoy X, Kampen H, Sweeney K, editors. Public health significance of urban pest. Geneva, Switzerland: World Health Organization; 2008 pp. 387–419.

[2] Aplin KP, Suzuki H, Chinen AA, Chesser RT, ten Have J, Donnellan SC, et al Multiple geographic origins of commensalism and complex dispersal history of black rats. PLoS One. 2011; 6(11): e26357 doi: 10.1371/journal.pone.0026357 22073158 10.1371/journal.pone.0026357 PMC3206810

[3] Pascal M. Rats. In: Simberloff D, Rejmanek M, editors. Encyclopedia of Biological Invasions. Berkeley and Los Angeles, California, USA: University of California Press; 2011 pp. 571–575.

[4] Courchamp F, Chapuis JL, Pascal M. Mammals invaders on islands: impact, control and control impact. Biol Rev Camb Philos Soc. 2003; 78: 347–383. 14558589 10.1017/s1464793102006061

[5] Pascal M, Lorvelec O, Vigne JD. Invasions biologiques et extinctions: 11 000 ans d'histoire des vertébrés en France. Belin & Quae ed Paris; 2006.

[6] Feng AYT, Himsworth CG. The secret life of the city rat: a review of the ecology of urban Norway and black rats (*Rattus norvegicus* and *Rattus rattus*). Urban Ecosyst. 2013; 17(1): 149–162.

[7] Centers for Disease Control and Prevention. Integrated pest management: conducting urban rodent surveys. Atlanta: US Department of Health and Human Services; 2006.

[8] Meerburg BG, Singleton GR, Kijlstra A. Rodent-borne diseases and their risks for public health. Crit Rev Microbiol. 2009; 35(3): 221–270. doi: 10.1080/10408410902989837 19548807 10.1080/10408410902989837

[9] Easterbrook JD, Shields T, Klein SL, Glass GE. Norway rat population in Baltimore, Maryland, 2004. Vector Borne Zoonotic Dis. 2005; 5(3): 296–299. doi: 10.1089/vbz.2005.5.296 16187901 10.1089/vbz.2005.5.296

[10] Smith MJ, Telfer S, Kallio ER, Burthe S, Cook AR, Lambin X, et al Host–pathogen time series data in wildlife support a transmission function between density and frequency dependence. Proc Natl Acad Sci USA. 2009; 106(19): 7905–7909. doi: 10.1073/pnas.0809145106 19416827 10.1073/pnas.0809145106 PMC2672915

[11] Boyle CM. Case of apparent resistance of *Rattus norvegicus* Berkenhout to anticoagulant poisons. Nature. 1960; 188(4749): 517–517.

[12] Pelz H-J, Rost S, Hünerberg M, Fregin A, Heiberg A-C, Baert K, et al The genetic basis of resistance to anticoagulants in rodents. Genetics. 2005; 170(4): 1839–1847. doi: 10.1534/genetics.104.040360 15879509 10.1534/genetics.104.040360 PMC1449767

[13] Easterbrook J, Kaplan J, Vanasco N, Reeves W, Purcell R, Kosoy M, et al A survey of zoonotic pathogens carried by Norway rats in Baltimore, Maryland, USA. Epidemiol Infect. 2007; 135(7): 1192–1199. doi: 10.1017/S0950268806007746 17224086 10.1017/S0950268806007746 PMC2870671

[14] Gardner-Santana LC, Norris DE, Fornadel CM, Hinson ER, Klein SL, Glass GE. Commensal ecology, urban landscapes, and their influence on the genetic characteristics of city-dwelling Norway rats (*Rattus norvegicus*). Mol Ecol. 2009; 18(13): 2766–2778. doi: 10.1111/j.1365-294X.2009.04232.x 19457177 10.1111/j.1365-294X.2009.04232.x PMC4118303

[15] Kajdacsi B, Costa F, Hyseni C, Porter F, Brown J, Rodrigues G, et al Urban population genetics of slum-dwelling rats (*Rattus norvegicus*) in Salvador, Brazil. Mol Ecol. 2013; 22(20): doi: 10.1111/mec.12455 24118116 10.1111/mec.12455 PMC3864905

[16] Firth C, Bhat M, Firth MA, Williams SH, Frye MJ, Simmonds P, et al Detection of zoonotic pathogens and characterization of novel viruses carried by commensal *Rattus norvegicus* in New York City. mBio. 2014; 5(5): e01933–14. doi: 10.1128/mBio.01933-14 25316698 10.1128/mBio.01933-14 PMC4205793

[17] Himsworth CG, Jardine CM, Parsons KL, Feng AYT, Patrick DM. The characteristics of wild rat (*Rattus* spp.) populations from an inner-city neighborhood with a focus on factors critical to the understanding of rat-associated zoonoses. PLoS One. 2014; 9(3): e91654 doi: 10.1371/journal.pone.0091654 24646877 10.1371/journal.pone.0091654 PMC3960114

[18] Davis DE, Emlen JT, Stokes AW. Studies on home range in the brown rat. J Mammal. 1948; 29(3): 207–225.

[19] Heiberg A-C, Sluydts V, Leirs H. Uncovering the secret lives of sewer rats (Rattus norvegicus): movements, distribution and population dynamics revealed by a capture–mark–recapture study. Wildl Res. 2012; 39(3): 202–219.

[20] Biek R, Real LA. The landscape genetics of infectious disease emergence and spread. Mol Ecol. 2010; 19(17): 3515–3531. doi: 10.1111/j.1365-294X.2010.04679.x 20618897 10.1111/j.1365-294X.2010.04679.x PMC3060346

[21] Guivier E, Galan M, Chaval Y, XuÉReb A, Ribas Salvador A, Poulle ML, et al Landscape genetics highlights the role of bank vole metapopulation dynamics in the epidemiology of Puumala hantavirus. Mol Ecol. 2011; 20(17): 3569–3583. doi: 10.1111/j.1365-294X.2011.05199.x 21819469 10.1111/j.1365-294X.2011.05199.x

[22] Richardson JL, Burak MK, Hernandez C, Shirvell JM, Mariani C, Carvalho‐Pereira TSA, et al Using fine‐scale spatial genetics of Norway rats to improve control efforts and reduce leptospirosis risk in urban slum environments. Evolutionary Applications. 2017; 10(4): 323–337. doi: 10.1111/eva.12449 28352293 10.1111/eva.12449 PMC5367079

[23] Mackenstedt U, Jenkins D, Romig T. The role of wildlife in the transmission of parasitic zoonoses in peri-urban and urban areas. Int J Parasitol Parasites Wildl. 2015; 4(1): 71–79. doi: 10.1016/j.ijppaw.2015.01.006 25830108 10.1016/j.ijppaw.2015.01.006 PMC4356871

[24] Bush AO, Fernández JC, Esch GW, Seed JR. Parasitism: the diversity and ecology of animal parasites. 1st ed Cambridge, UK: Cambridge University press; 2001.

[25] Lorvelec O, Pascal M. French attempts to eradicate non-indigenous mammals and their consequences for native biota. Biol Invasions. 2005; 7(1): 135–140.

[26] McGuire B, Pizzuto T, Bemis WE, Getz LL. General ecology of a rural population of Norway rats (*Rattus norvegicus*) based on intensive live trapping. Am Midl Nat. 2006; 155(1): 221–236.

[27] Berthier K, Charbonnel N, Galan M, Chaval Y, Cosson JF. Migration and recovery of the genetic diversity during the increasing density phase in cyclic vole populations. Mol Ecol. 2006; 15(9): 2665–2676. doi: 10.1111/j.1365-294X.2006.02959.x 16842435 10.1111/j.1365-294X.2006.02959.x

[28] Grandemange A, Lasseur R, Longin-Sauvageon C, Benoit E, Berny P. Distribution of VKORC1 single nucleotide polymorphism in wild *Rattus norvegicus* in France. Pest Manag Sci. 2010; 66(3): 270–276. doi: 10.1002/ps.1869 19890940 10.1002/ps.1869

[29] Fourel I, Damin-Pernik M, Benoit E, Lattard V. Core-shell LC–MS/MS method for quantification of second generation anticoagulant rodenticides diastereoisomers in rat liver in relationship with exposure of wild rats. J Chromatogr B. 2017; 1041–1042: 120–132. 10.1016/j.jchromb.2016.12.028 28033586

[30] Galan M, Guivier E, Caraux G, Charbonnel N, Cosson J-F. A 454 multiplex sequencing method for rapid and reliable genotyping of highly polymorphic genes in large-scale studies. BMC Genomics. 2010; 11: 296–296. doi: 10.1186/1471-2164-11-296 20459828 10.1186/1471-2164-11-296 PMC2876125

[31] Kramski M, Meisel H, Klempa B, Krüger DH, Pauli G, Nitsche A. Detection and typing of human pathogenic hantaviruses by real-time reverse transcription-PCR and pyrosequencing. Clin Chem. 2007; 53(11): 1899–1905. doi: 10.1373/clinchem.2007.093245 17717126 10.1373/clinchem.2007.093245

[32] Ninove L, Domart Y, Vervel C, Voinot C, Salez N, Raoult D, et al Cowpox virus transmission from pet rats to humans, France. Emerg Infect Dis. 2009; 15(5): 781–784. doi: 10.3201/eid1505.090235 19402968 10.3201/eid1505.090235 PMC2686997

[33] Homberger FR, Smith AL, Barthold SW. Detection of rodent coronaviruses in tissues and cell cultures by using polymerase chain reaction. J Clin Microbiol. 1991; 29(12): 2789–2793. 1661745 10.1128/jcm.29.12.2789-2793.1991 PMC270434

[34] Rose N, Lunazzi A, Dorenlor V, Merbah T, Eono F, Eloit M, et al High prevalence of Hepatitis E virus in French domestic pigs. Comp Immunol Microbiol Infect Dis. 2011; 34(5): 419–427. doi: 10.1016/j.cimid.2011.07.003 21872929 10.1016/j.cimid.2011.07.003

[35] Johne R, Plenge-Bönig A, Hess M, Ulrich RG, Reetz J, Schielke A. Detection of a novel Hepatitis E-like virus in faeces of wild rats using a nested broad-spectrum RT-PCR. J Gen Virol. 2010; 91(3): 750–758. 19889929 10.1099/vir.0.016584-0

[36] Bonnet S, Jouglin M, Malandrin L, Becker C, Agoulon A, L'Hostis M, et al Transstadial and transovarial persistence of *Babesia divergens* DNA in *Ixodes ricinus* ticks fed on infected blood in a new skin-feeding technique. Parasitology. 2007; 134(02): 197–207. 17076925 10.1017/S0031182006001545

[37] Parola P, Roux V, Camicas J-L, Baradji I, Brouqui P, Raoult D. Detection of ehrlichiae in African ticks by polymerase chain reaction. Trans R Soc Trop Med Hyg. 2000; 94(6): 707–708. 11198664 10.1016/s0035-9203(00)90243-8

[38] Marconi RT, Garon CF. Development of polymerase chain reaction primer sets for diagnosis of Lyme disease and for species-specific identification of Lyme disease isolates by 16S rRNA signature nucleotide analysis. J Clin Microbiol. 1992; 30(11): 2830–2834. 1280643 10.1128/jcm.30.11.2830-2834.1992 PMC270537

[39] Norman AF, Regnery R, Jameson P, Greene C, Krause DC. Differentiation of *Bartonella*-like isolates at the species level by PCR-restriction fragment length polymorphism in the citrate synthase gene. J Clin Microbiol. 1995; 33(7): 1797–803. 7545181 10.1128/jcm.33.7.1797-1803.1995 PMC228273

[40] Michelet L, Delannoy S, Devillers E, Umhang G, Aspan A, Juremalm M, et al High-throughput screening of tick-borne pathogens in Europe. Front Cell Infect Microbiol. 2014; 4: 103 doi: 10.3389/fcimb.2014.00103 25120960 10.3389/fcimb.2014.00103 PMC4114295

[41] Regnery RL, Spruill CL, Plikaytis BD. Genotypic identification of rickettsiae and estimation of intraspecies sequence divergence for portions of two rickettsial genes. J Bacteriol. 1991; 173(5): 1576–1589. 1671856 10.1128/jb.173.5.1576-1589.1991 PMC207306

[42] Higgins JA, Hubalek Z, Halouzka J, Elkins KL, Sjostedt A, Shipley M, et al Detection of *Francisella tularensis* in infected mammals and vectors using a probe-based polymerase chain reaction. Am J Trop Med Hyg. 2000; 62(2): 310–8. 10813490 10.4269/ajtmh.2000.62.310

[43] Mérien F, Amouriaux P, Pérolat P, Baranton G, Saint-Girons I. Polymerase chain reaction for detection of *Leptospira* spp. in clinical sample. J Clin Microbiol. 1992; 30(9): 2219–2224. 1400983 10.1128/jcm.30.9.2219-2224.1992 PMC265482

[44] Postic D, Riquelme-Sertour N, Merien F, Pérolat P, Baranton G. Interest of partial 16S rDNA gene sequences to resolve heterogeneities between *Leptospira* collections: application to *L*. *meyeri*. Res Microbiol. 2000; 151(5): 333–341. 10919513 10.1016/s0923-2508(00)00156-x

[45] Beaucournu JC, Launay H. Les puces (Siphonaptera): De France et du Bassin mediterraneen occidental (Faune de France). Paris, France: Fédération française des sociétés de sciences naturelles; 1990.

[46] Ribas A, Bellocq J, Ros A, Ndiaye P, Miquel J. Morphometrical and genetic comparison of two nematode species: *H*. *spumosa* and *H*. *dahomensis* (Nematoda, Heterakidae). Acta Parasitol. 2013; 58(3): 389–398. doi: 10.2478/s11686-013-0156-4 23990438 10.2478/s11686-013-0156-4

[47] del Rosario Robles M, Navone GT, Villafañe IEG. New morphological details and first records of *Heterakis spumosa* and *Syphacia muris* from Argentina. Comp Parasitol. 2008; 75(1): 145–149.

[48] Durette-Desset MC. Le genre *Nippostrongylus* Lane, 1923, (Nématode—Héligmosomatidé) [French]. Ann Parasitol Hum Comp. 1970; 45(6): 818–821. 10.1051/parasite/1970456815 5535153

[49] Hugot JP, Quentin JC. Etude morphologique de six espèces nouvelles ou peu connues appartenant au genre *Syphacia* (Oxyuridae, Nematoda), parasites de Rongeurs, Cricétidés et Muridés [French]. Bull Mus Hist. 1985; 4(7): 383–400.

[50] Anderson RC, Chabaud AG, Willmott S. Keys to the Nematode Parasites of Vertebrates: Archival volume. Wallingford (UK): CAB International; 2009.

[51] Casanova JC, Santalla F, Durand P, Vaucher C, Feliu C, Renaud F. Morphological and genetic differentiation of *Rodentolepis straminea* (Goeze, 1752) and *Rodentolepis microstoma* (Dujardin, 1845) (Hymenolepididae). Parasitol Res. 2001; 87(6): 439–444. 11411941 10.1007/s004360100379

[52] Makarikov AA, Tkach VV. Two new species of *Hymenolepis* (Cestoda: Hymenolepididae) from *Spalacidae* and Muridae (Rodentia) from eastern Palearctic. Acta Parasitol. 2013; 58(1): 37–49. doi: 10.2478/s11686-013-0115-0 23377911 10.2478/s11686-013-0115-0

[53] Gardner SL. Helminth parasites of *Thomomys bulbivorus* (Richardson) (Rodentia: Geomyidae), with the description of a new species of *Hymenolepis* (Cestoda). Can J Zool. 1985; 63(6): 1463–1469.

[54] Khalil LF, Bray RA, Jones A. Keys to the cestode parasites of vertebrates. Wallingford, UK: CAB International; 1994.

[55] Pojmanska T. Superfamily Brachylaimoidea Joyeux and Foley In: David Ian Gibson AJ, ‎Rodney Alan Bray, editor. Keys to the Trematoda. CAB International ed. London, UK: CAB International and The Natural History Museum; 2002 pp. 31–45.

[56] Nelson L, Clark FW. Correction for sprung traps in catch/effort calculations of trapping results. J Mammal. 1973; 54(1): 295–298.

[57] Theuerkauf J, Rouys S, Jourdan H. Efficiency of a new reverse-bait trigger snap trap for invasive rats and a new standardised abundance index. Ann Zool Fenn. 2011; 48: 308–318.

[58] QGIS Development Team. QGIS Geographic Information System. Open Source Geospatial Foundation Project. v. 2.16.3; 2016.

[59] R Development Core Team. R: A language and environment for statistical computing. R Foundation for Statistical Computing, Vienna, Austria; 2016; Available from: http://www.R-project.org.

[60] Nei M. Estimation of average heterozygosity and genetic distance from a small number of individuals. Genetics. 1978; 89(3): 583–590. 17248844 10.1093/genetics/89.3.583 PMC1213855

[61] Weir BS, Cockerham CC. Estimating F-statistics for the analysis of population structure. Evolution. 1984; 38(6): 1358–1370. doi: 10.1111/j.1558-5646.1984.tb05657.x 28563791 10.1111/j.1558-5646.1984.tb05657.x

[62] Belkhir K, Borsa P, Chikhi L, Raufaste N, Bonhomme F. GENETIX, logiciel sous WindowsTM pour la génétique des populations. v.; 2001.

[63] Raymond M, Rousset F. GENEPOP (Version 1.2): Population genetics software for exact tests and ecumenicism. J Hered. 1995; 86(3): 248–249.

[64] Benjamini Y, Hochberg Y. Controlling the false discovery rate: a practical and powerful approach to multiple testing. Journal of the Royal Statistical Society Series B (Methodological). 1995; 57(1): 289–300.

[65] Hall SA, Kaufman JS, Ricketts TC. Defining urban and rural areas in U.S. epidemiologic studies. Journal of Urban Health. 2006; 83(2): 162–175. doi: 10.1007/s11524-005-9016-3 16736366 10.1007/s11524-005-9016-3 PMC2527174

[66] Barnett SA. The rat: a study in behaviour. Canberra: Australian National University Press; 1976.

[67] Wang J. An estimator for pairwise relatedness using molecular markers. Genetics. 2002; 160(3): 1203–1215. 11901134 10.1093/genetics/160.3.1203 PMC1462003

[68] Hardy OJ, Vekemans X. spagedi: a versatile computer program to analyse spatial genetic structure at the individual or population levels. Mol Ecol Notes. 2002; 2(4): 618–620.

[69] Piry S, Alapetite A, Cornuet J-M, Paetkau D, Baudouin L, Estoup A. GENECLASS2: A software for genetic assignment and first-generation migrant detection. J Hered. 2004; 95(6): 536–539. doi: 10.1093/jhered/esh074 15475402 10.1093/jhered/esh074

[70] Paetkau D, Slade R, Burden M, Estoup A. Genetic assignment methods for the direct, real-time estimation of migration rate: a simulation-based exploration of accuracy and power. Mol Ecol. 2004; 13(1): 55–65. 14653788 10.1046/j.1365-294x.2004.02008.x

[71] Chapuis M-P, Estoup A. Microsatellite null alleles and estimation of population differentiation. Mol Biol Evol. 2007; 24(3): 621–631. doi: 10.1093/molbev/msl191 17150975 10.1093/molbev/msl191

[72] Cornuet JM, Luikart G. Description and power analysis of two tests for detecting recent population bottlenecks from allele frequency data. Genetics. 1996; 144(4): 2001–2014. 8978083 10.1093/genetics/144.4.2001 PMC1207747

[73] Piry S, Luikart G, Cornuet JM. Computer note. BOTTLENECK: a computer program for detecting recent reductions in the effective size using allele frequency data. J Hered. 1999; 90(4): 502–503.

[74] Wasserman S, Faust K. Social network analysis: methods and applications. Cambridge; New York, NY: Cambridge University Press; 1994.

[75] Vaumourin E, Vourc'h G, Telfer S, Lambin X, Salih D, Seitzer U, et al To be or not to be associated: power study of four statistical modeling approaches to identify parasite associations in cross-sectional studies. Front Cell Infect Microbiol. 2014; 4: 62 doi: 10.3389/fcimb.2014.00062 24860791 10.3389/fcimb.2014.00062 PMC4030204

[76] Csardi G, Nepusz T. The igraph software package for complex network research. InterJournal. 2006; Complex Systems: 1695.

[77] Directive 2010/63/EU of the European parliament and of the Council of 22 September 2010 on the protection of animals used for scientific purposes, (2010).

[78] Turton JA, Hawkey CM, Hart MG, Gwynne J, Hicks RM. Age-related changes in the haematology of female F344 rats. Lab Anim. 1989; 23(4): 295–301. doi: 10.1258/002367789780746006 2811267 10.1258/002367789780746006

[79] Kampfmann I, Bauer N, Johannes S, Moritz A. Differences in hematologic variables in rats of the same strain but different origin. Vet Clin Pathol. 2012; 41(2): 228–234. doi: 10.1111/j.1939-165X.2012.00427.x 22551195 10.1111/j.1939-165X.2012.00427.x

[80] Slatkin M. Linkage disequilibrium—understanding the evolutionary past and mapping the medical future. Nat Rev Genet. 2008; 9(6): 477–485. doi: 10.1038/nrg2361 18427557 10.1038/nrg2361 PMC5124487

[81] Clapperton BK. A review of the current knowledge of rodent behaviour in relation to control devices Wellington (New Zealand): Science & Technical Publishing Department of Conservation; 2006.

[82] Storz JF, Bhat HR, Kunz TH. Genetic consequences of polygyny and social structure in an Indian fruit bat, *Cynopterus sphinx*. I. Inbreeding, outbreeding, and population subdivision. Evolution. 2001; 55(6): 1215–1223. 11475057 10.1111/j.0014-3820.2001.tb00641.x

[83] Calhoun JB. The ecology and sociology of the Norway rat. Bethesda, Maryland: U.S. Dept. of Health, Education, and Welfare, Public Health Service; 1963.

[84] Greenwood PJ. Mating systems, philopatry and dispersal in birds and mammals. Anim Behav. 1980; 28(4): 1140–1162.

[85] Sommer S. The importance of immune gene variability (MHC) in evolutionary ecology and conservation. Front Zool. 2005; 2: 16–16. doi: 10.1186/1742-9994-2-16 16242022 10.1186/1742-9994-2-16 PMC1282567

[86] Lenz TL, Wells K, Pfeiffer M, Sommer S. Diverse MHC IIB allele repertoire increases parasite resistance and body condition in the Long-tailed giant rat (*Leopoldamys sabanus*). BMC Evol Biol. 2009; 9(1): 1–13. 19930637 10.1186/1471-2148-9-269 PMC2788554

[87] Pagès M, Bazin E, Galan M, Chaval Y, Claude J, herbreteau V, et al Cytonuclear discordance among Southeast Asian black rats (*Rattus rattus* complex). Mol Ecol. 2013; 22(4): 1019–1034. doi: 10.1111/mec.12149 23278980 10.1111/mec.12149

[88] McKinney ML. Urbanization, biodiversity, and conservation. BioScience. 2002; 52(10): 883–890.

[89] Ellegren H, Galtier N. Determinants of genetic diversity. Nat Rev Genet. 2016; 17(7): 422–433. doi: 10.1038/nrg.2016.58 27265362 10.1038/nrg.2016.58

[90] Vandenbroucke V, Bousquet-Melou A, De Backer P, Croubels S. Pharmacokinetics of eight anticoagulant rodenticides in mice after single oral administration. J Vet Pharmacol Ther. 2008; 31(5): 437–445. doi: 10.1111/j.1365-2885.2008.00979.x 19000263 10.1111/j.1365-2885.2008.00979.x

[91] Vaumourin E, Vourc’h G, Gasqui P, Vayssier-Taussat M. The importance of multiparasitism: examining the consequences of co-infections for human and animal health. Parasit Vectors. 2015; 8(1): 1–13. 26482351 10.1186/s13071-015-1167-9 PMC4617890

[92] Tollenaere C, Susi H, Laine A-L. Evolutionary and epidemiological implications of multiple infection in plants. Trends Plant Sci. 2016; 21(1): 80–90. doi: 10.1016/j.tplants.2015.10.014 26651920 10.1016/j.tplants.2015.10.014

[93] Stahl W. Studies on the life cycle of *Syphacia muris*, the rat pinworm. Keio J Med. 1963; 12(2): 55–60. 13978720 10.2302/kjm.12.55

[94] Klimpel S, Förster M, Schmahl G. Parasites of two abundant sympatric rodent species in relation to host phylogeny and ecology. Parasitol Res. 2006; 100(4): 867–875. doi: 10.1007/s00436-006-0368-8 17120043 10.1007/s00436-006-0368-8

[95] Šnábel V, Utsuki D, Kato T, Sunaga F, Ooi H-K, Gambetta B, et al Molecular identification of Heterakis spumosa obtained from brown rats (*Rattus norvegicus*) in Japan and its infectivity in experimental mice. Parasitol Res. 2014; 113(9): 3449–3455. doi: 10.1007/s00436-014-4014-6 24997621 10.1007/s00436-014-4014-6

[96] Meade TM, Watson J. Characterization of rat pinworm (*Syphacia muris*) epidemiology as a means to increase detection and elimination. J Am Assoc Lab Anim Sci. 2014; 53(6): 661–667. 25650973 PMC4253580

[97] Psaroulaki A, Antoniou M, Toumazos P, Mazeris A, Ioannou I, Chochlakis D, et al Rats as indicators of the presence and dispersal of six zoonotic microbial agents in Cyprus, an island ecosystem: a seroepidemiological study. Trans R Soc Trop Med Hyg. 2010; 104(11): 733–739. doi: 10.1016/j.trstmh.2010.08.005 20870259 10.1016/j.trstmh.2010.08.005

[98] Lehmann JS, Matthias MA, Vinetz JM, Fouts DE. Leptospiral pathogenomics. Pathogens. 2014; 3(2): 280–308. doi: 10.3390/pathogens3020280 25437801 10.3390/pathogens3020280 PMC4243447

[99] Desvars A, Naze F, Vourc'h G, Cardinale E, Picardeau M, Michault A, et al Similarities in *Leptospira* serogroup and species distribution in animals and humans in the Indian Ocean island of Mayotte. Am J Trop Med Hyg. 2012; 87(1): 134–140. doi: 10.4269/ajtmh.2012.12-0102 22764304 10.4269/ajtmh.2012.12-0012 PMC3391038

[100] Villanueva SYAM, Ezoe H, Baterna RA, Yanagihara Y, Muto M, Koizumi N, et al Serologic and molecular studies of *Leptospira* and leptospirosis among rats in the Philippines. Am J Trop Med Hyg. 2010; 82(5): 889–898. doi: 10.4269/ajtmh.2010.09-0711 20439972 10.4269/ajtmh.2010.09-0711 PMC2861393

[101] Scialfa E, Bolpe J, Bardón JC, Ridao G, Gentile J, Gallicchio O. Isolation of *Leptospira interrogans* from suburban rats in Tandil, Buenos Aires, Argentina. Rev Argent Microbiol. 2010; 42(2): 126–128. doi: 10.1590/S0325-75412010000200012 20589335 10.1590/S0325-75412010000200012

[102] Guernier V, Lagadec E, Cordonin C, Le Minter G, Gomard Y, Pagès F, et al Humanleptospirosis on Reunion island, Indian Ocean: are rodents the (only) ones to blame? PLoS Negl Trop Dis. 2016; 10(6): e0004733 doi: 10.1371/journal.pntd.0004733 27294677 10.1371/journal.pntd.0004733 PMC4905629

[103] Barnett SA, Spencer MM. Experiments on the food preferences of wild rats (*Rattus norvegicus* Berkenhout). J Hyg. 1953; 51(1): 16–34. 13044928 10.1017/s002217240001545x PMC2217682

[104] Smith HM, Dickman CR, Banks PB. Nest predation by commensal rodents in urban bushland remnants. PLoS One. 2016; 11(6): e0156180 doi: 10.1371/journal.pone.0156180 27295091 10.1371/journal.pone.0156180 PMC4905641

[105] Abdelkrim J, Pascal M, Calmet C, Samadi S. Importance of assessing population genetic structure before eradication of invasive species: Examples from insular Norway rat populations. Conserv Biol. 2005; 19(5): 1509–1518.

[106] Singleton GR, Hinds LA, Leirs H, Zhang Z. Ecologically-based management of rodent pests. Monograph No. 59 Canbera: Australian Centre for International Agricultural Research (ACIAR); 1999.

[107] Traweger D, Travnitzky R, Moser C, Walzer C, Bernatzky G. Habitat preferences and distribution of the brown rat (*Rattus norvegicus* Berk.) in the city of Salzburg (Austria): implications for an urban rat management. J Pest Sci. 2006; 79(3): 113–125.

