# Supplementary material for: Population genetics, community of parasites, and resistance to rodenticides in an urban brown rat (Rattus norvegicus) population
Source: PLoS One. 2017 Sep 8;12(9):e0184015. doi: 10.1371/journal.pone.0184015 (PMC5590879; doi:10.1371/journal.pone.0184015)
Supplement: S1 Table — Median and range (minimum–maximum) are given for the quantitative data, number and percentage for the qualitative data. One animal on site 1 was not sexed, weight data were missing for two females and two males on site 1, which therefore could not have been categorized as young or adult. (PDF) [file pone.0184015.s002.pdf]

S1 Table. Summary of the results per site and sex. Median and range (minimum – maximum) are given for the quantitative data, number and percentage for the qualitative data. One animal on site 1 was not sexed, weight data were missing for two females and two males on site 1, which therefore could not have been categorized as young or adult.

	**Site 1**		**Site 2**	
	**Males**	**Females**	**Males**	**Females**
**Total number of individuals**	45	34	4	2
Number of young (%)	20 (44.4)	13 (38.2)	2 (50.0)	0
**Body measurements** (median, min. – max.)				
Body weight alive (g)	212.0 (49.0 – 419.0)	191.5 (55.0 – 452.0)	233.0 (136.0 – 292.0)	195.0 (186.0 – 204.0)
Carcass weight (g)	167.0 (42.0 – 356.0)	159.0 (43.0 – 341.0)	195.0 (114.0 – 258.0)	137.5 (109.0 – 166.0)
Body length (mm)	197 (112 – 280)	194 (100 – 255)	206 (175 – 225)	191 (177 – 205)
Tail length (mm)	148 (94.– 257)	148 (104 – 210)	163 (150 – 175)	147 (147 – 148)
**Haematology** (median, min. – max.)				
WBC count (x1,000/mm^3)	6.68 (1.26 – 19.62)	8.40 (1.84 – 42.93)	8.82 (7.27 – 20.9)	9.68 (7.97 – 11.38)
RBC count (x1,000/mm^3)	8.36 (2.11 – 11.86)	7.96 (3.55 – 11.13)	9.40 (8.29 – 9.53)	7.92 (7.02 – 8.81)
Platelet count (x1,000/mm^3)	483.0 (47.0 – 972.0)	642.0 (42.0 – 1004.0)	517.5 (448.0 – 614.0)	682.0 (640.0 – 724.0)
**Population genetics**				
Number migrants (%)	2 (4.4)	0	2 (50.0)	0
***VKORC1* genotype** (number, %)				
-/-	9 (20.0)	4 (11.8)	2 (50.0)	1 (50.0)
Y139F/-	20 (44.4)	17 (50.0)	2 (50.0)	1 (50.0)
Y139F/Y139F	16 (35.6)	13 (38.2)	0	0 (50.0)

(S1 Table continued)	**Site 1**		**Site 2**	
	**Males**	**Females**	**Males**	**Females**
**AVK residues** (Number of positive, %)				
Coumatetralyl	1 (2.2)	1 (2.9)	0	0
Warfarin	0	0	0	0
Chlorophacinone	7 (15.6)	6 (17.6)	0	0
Bromadiolone	6 (13.3)	4 (11.8)	1 (25.0)	2 (100)
Difenacoum	17 (37.8)	15 (44.1)	0	0
Brodifacoum	15 (33.3)	13 (38.2)	0	0
Flocoumafen	0	0	0	0
Difethialone	9 (20.0)	9 (26.5)	0	0
**Microparasite** (Number of positive, %)				
Seoul hantavirus	0	0	0	0
Orthopoxvirus	0	0	0	0
Coronavirus	0	0	0	0
Hepatitis E virus	0	0	0	0
Trypanosomes	17 (37.8)	10 (29.4)	2 (50.0)	0
Babesia sp./*Theileria* sp.	0	0	0	0
Anaplasma sp. and *Ehrlichia* sp.	0	0	0	0
Borrelia burgdorferi s.l.	0	0	0	0
Coxiella burnetii	0	0	0	0
Bartonella sp.	23 (51.1)	16 (47.1)	0	0
Rickettsia sp.	0	1 (2.9)	0	0
Francisella tularensis	3 (6.7)	1 (2.9)	0	0
Leptospira sp.	11 (24.4)	6 (17.6)	0	0

(S1 Table continued)	**Site 1**		**Site 2**	
	**Males**	**Femelles**	**Males**	**Femelles**
**Macroparasite** (Number of positive, %)				
Fleas (all species)	15 (33.3)	19 (55.9)	3 (75.0)	0
Heterakis spumosa	20 (44.4)	23 (67.7)	4 (100)	2 (100.0)
Syphacia muris	28 (62.2)	15 (44.1)	1 (25.0)	0
Nippostrongylus brasiliensis	0	0	3 (75.0)	2 (100.0)
Rodentolepis microstoma	5 (11.1)	1 (2.9)	0	0
Hymenolepis diminuta	1 (2.2)	0	0	0
Brachylaima sp.	0	1 (2.9)	0	0
Capillaridae	0	0	1 (25.0)	0
**Multiparasitism**				
Total number of parasites (median, min., max.)	3.0 (0.0 – 6.0)	3.0 (3.0 – 5.0)	3.0 (3.0 – 5.0)	2 (2 – 2)
