# Supplementary material for: Population genetics, community of parasites, and resistance to rodenticides in an urban brown rat (Rattus norvegicus) population
Source: PLoS One. 2017 Sep 8;12(9):e0184015. doi: 10.1371/journal.pone.0184015 (PMC5590879; doi:10.1371/journal.pone.0184015)
Supplement: S2 Table — (PDF) [file pone.0184015.s003.pdf]

S2 Table. Setting and results of the trapping sessions, 10–21 January 2011, on sites 1 and 2 in Chanteraines park (Hauts-de-Seine, France).

	**Number of traps**							
	**Set**		**Sprung with no catch**		**Non-target species**[1]		**Rat trapped**[2]	
**Date**	**Day**	**Night**	**Day**	**Night**	**Day**	**Night**	**Day**	**Night**
**Site 1**								
10/01/2011	0	296	0	8	0	1	0	2
11/01/2011	296	297	20	5	12	1	7	9
12/01/2011	314	332	25	6	10	0	6	3
13/01/2011	356	357	35	8	7	3	13	9
14/01/2011	364	376	21	2	18	2	12	1
15/01/2011	376	376	64	28	13	7	6	1
16/01/2011	376	376	60	29	11	7	4	2
17/01/2011	376	376	68	3	11	5	0	0
18/01/2011	376	376	40	31	13	7	3	0
19/01/2011	376	376	23	14	20	6	1	0
20/01/2011	376	376	65	14	10	9	0	1
**Site 2**								
10/01/2011	0	296	0	2	0	0	0	0
11/01/2011	310	310	55	5	10	2	1	0
12/01/2011	310	310	84	10	17	3	0	0
13/01/2011	310	310	100	7	25	7	2	0
14/01/2011	325	325	117	20	29	13	0	1
17/01/2011	0	88	-	13	0	3	0	0
18/01/2011	88	0	22	0	1	0	0	0
19/01/2011	88	88	45	5	11	5	0	0
20/01/2011	88	0	24	0	7	0	1	0

[1] Non target species caught were: 31 tits *Parus* spp.,17 robins *Erithacus rubecula*, two house sparrows *Passer domesticus*, one dunnock *Prunella modularis*), five moorhens *Gallinula chloropus*, four doves *Columba oenas*, and three magpies *Pica pica*, 107 wood mice *Apodemus sylvaticus*, three hedgehogs *Erinaceus europaeus*.

[2] One rat has been non conventionally caught by hands and does not appear in this table.
