# Supplementary material for: Population genetics, community of parasites, and resistance to rodenticides in an urban brown rat (Rattus norvegicus) population
Source: PLoS One. 2017 Sep 8;12(9):e0184015. doi: 10.1371/journal.pone.0184015 (PMC5590879; doi:10.1371/journal.pone.0184015)
Supplement: S3 Table — Adjusted odds ratios and 95% confidence intervals for pathogen occurrence in R. norvegicus from Chanteraines park calculated using the best-fitted general linear model identified using a stepwise backward selection on the Akaike Information Criterion. (PDF) [file pone.0184015.s004.pdf]

S3 Table. Model 2 results. Adjusted odds ratios and 95% confidence intervals for pathogen occurrence in *R. norvegicus* from Chanteraines park calculated using the best-fitted general linear model identified using a stepwise backward selection on the Akaike Information Criterion.

Variables	Categories	Flea	*S. muris*	*H. spumosa*	*Leptospira sp.*
Site	Site 1	/	Ref	/	Not included
	Site 2	/	0.03 (0.00–0.48)*	/	Not included
Sex	Female	Ref	Ref	Ref	/
	Male	0.40 (0.13–1.17)	3.16 (0.98–11.18)	0.20 (0.04–0.80)*	/
Age	Adult	/	Ref	Ref	Ref
	Subadult	/	21.34 (3.58–414.26)*	0.50 (0.12–2.13)	
	Juvenile	/	3.76 (0.84–21.24)	0.02 (0.00–0.18)*	
WBC count (x 1,000/mm^3)	< 6.6	Ref	/	Ref	Ref
	[6.6–9.2[	0.27 (0.07–0.93)*	/	8.52 (2.06–43.23)*	1.07 (0.28–4.13)
	≥ 9.2	0.29 (0.08–1.00)	/	3.23 (0.81–13.89)	0.12 (0.00–0.80)*
RBC count (x 1,000/mm^3)	< 7.0	/	/	/	/
	[7.0–8.8[	/	/	/	/
	≥ 8.8	/	/	/	/
Platelet count (x 1,000/mm^3)	< 452.0	/	/	/	/
	[452.0–645.0[	/	/	/	/
	≥ 645.0	/	/	/	/

No model can be fitted for *Trypanosoma* sp. and *Bartonella* sp. occurrence with the explanatory variables considered. “Not included” means that the variable was not included in the GLM because the contingency table between the dependent variable and an explanatory variable presented zero cells. Significant adjusted odds ratio are shown by asterisks.
