# Supplementary figures and images for: Population genetics, community of parasites, and resistance to rodenticides in an urban brown rat (Rattus norvegicus) population
Source: PLoS One. 2017 Sep 8;12(9):e0184015. doi: 10.1371/journal.pone.0184015 (PMC5590879; doi:10.1371/journal.pone.0184015)

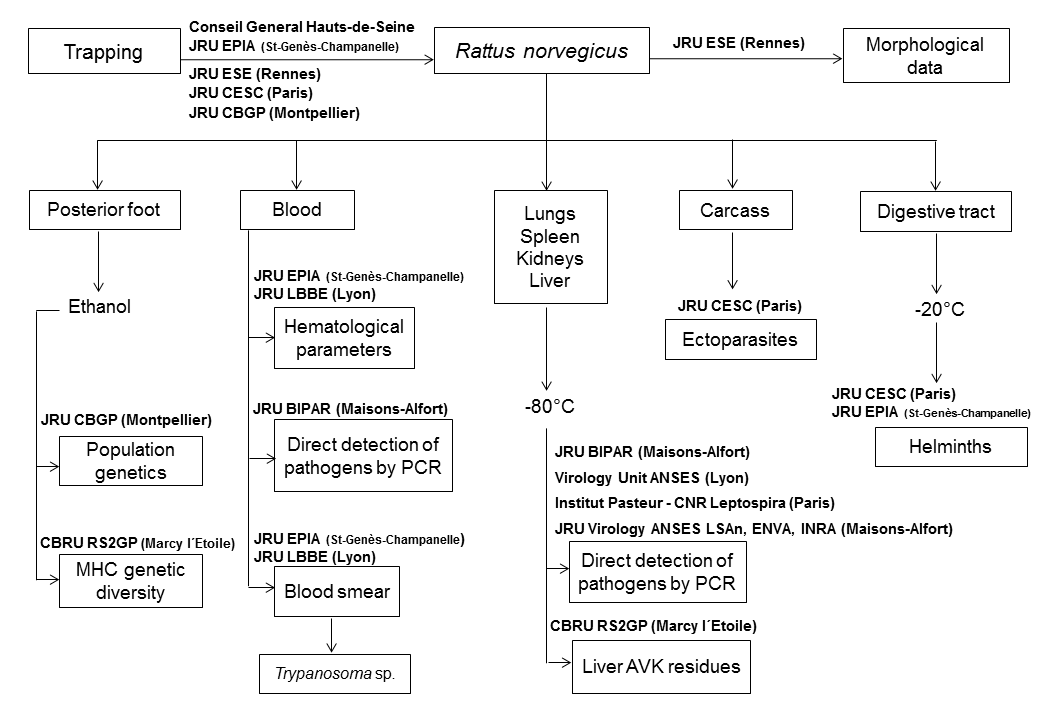

Supplement: S1 Fig — (TIF) [file pone.0184015.s005.tif]

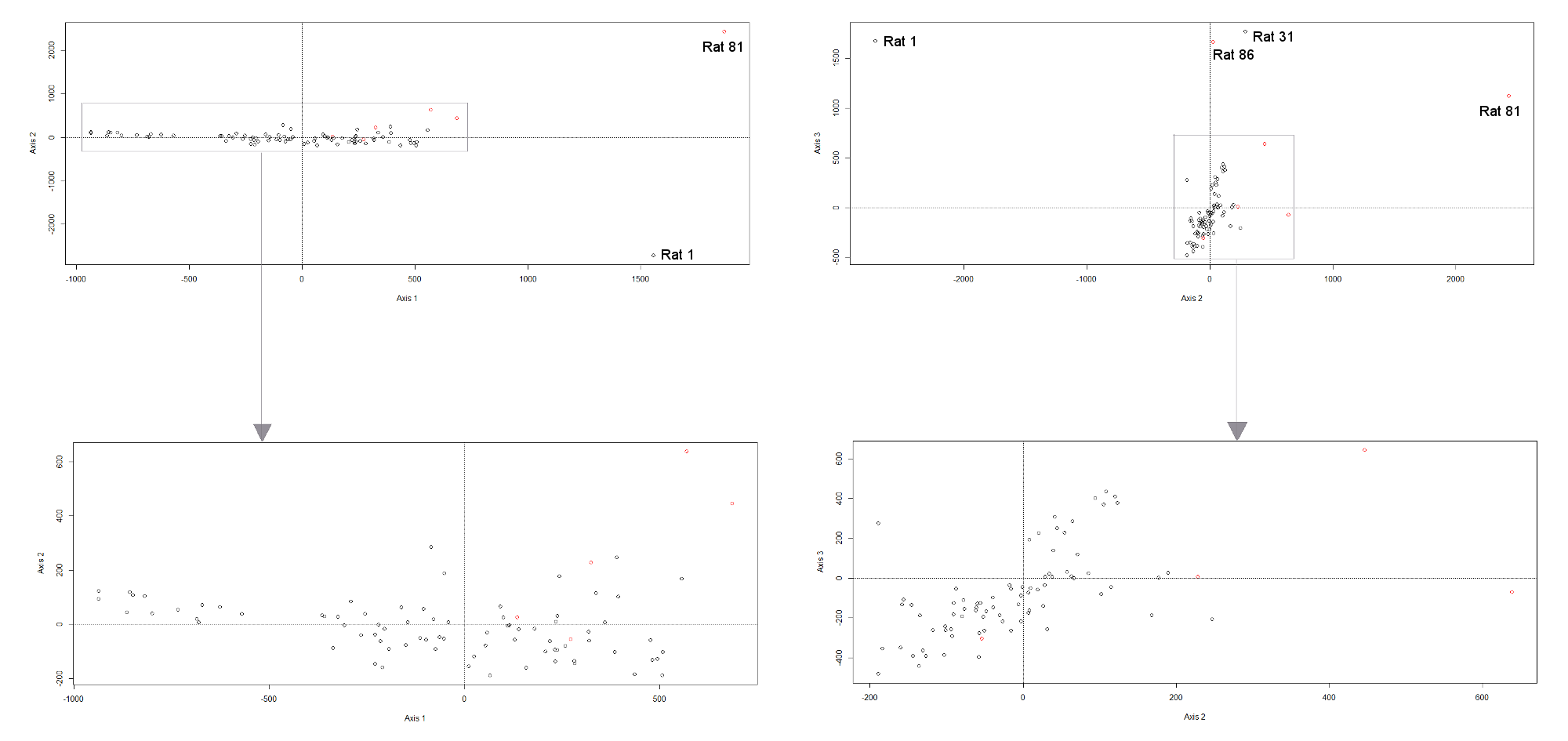

Supplement: S2 Fig — Individuals from site 1 are in black, red signs represent individuals from site 2. Identification names of the four individuals outside the main scatter plots are written, they correspond to the four migrants. (TIF) [file pone.0184015.s006.tif]

L(K) (mean  $\pm$  SD)

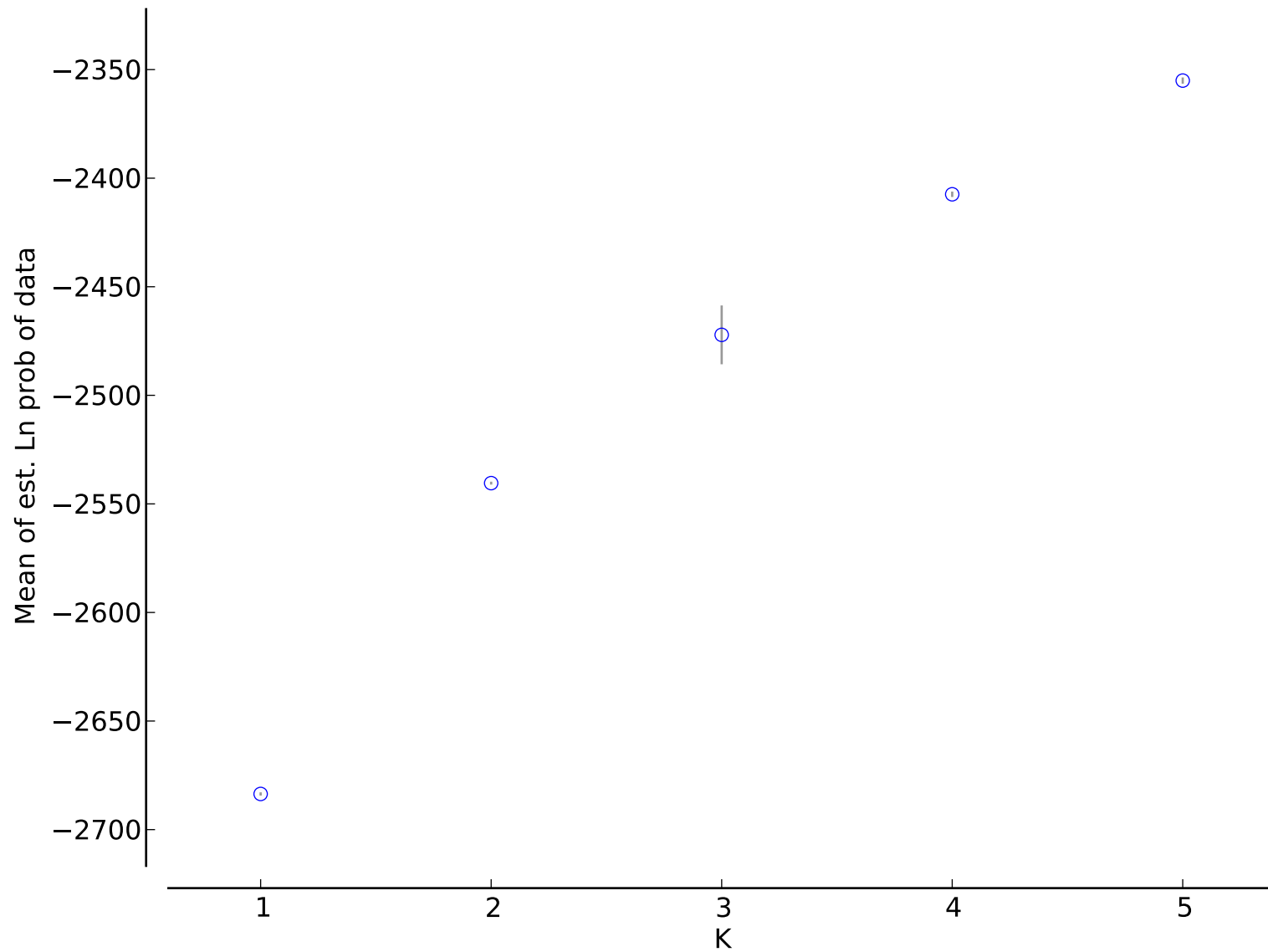

Supplement: S3 Fig — Plot of mean likelihood L(K) and variance per K value from Structure (86 individuals genotyped for 13 polymorphic microsatellite loci). (PDF) [file pone.0184015.s007.pdf]
